# Using simulation to disambiguate transducer and noise

**DOI:** 10.1167/jov.26.10.12

**Published:** 2026-10-13

**Authors:** Guillermo Aguilar, Marianne Maertens, Joris Vincent

**Affiliations:** 1 Computational Psychology, Technische Universität Berlin, Berlin, Germany

**Keywords:** sensitivity, appearance, transducer, discrimination, MLDS, scaling

## Abstract

The relationship between perceived stimulus magnitude and physical stimulus intensity has traditionally been probed through two largely separate experimental traditions: near-threshold measures of sensitivity and suprathreshold measures of appearance. Reconciling both approaches has proven difficult because they rely on different theoretical assumptions and involve different experimental techniques. Here we argue that the empirical relationship between sensitivity and appearance cannot be resolved by experiment alone and propose a simulation-based approach, which requires explicitly specifying the transducer, the noise model, and the observer’s decision rule. Within this framework, we evaluate whether maximum likelihood difference scaling (MLDS) can recover a known transducer from simulated data, especially for the critical cases in which different transducer and noise combinations produce identical discrimination performance. We simulate MLDS experiments for logarithmic and power-law transducers combined with additive and multiplicative noise at magnitudes representative of psychophysical data. MLDS recovers compressive, linear, and moderately expansive transducers accurately for both noise types. Systematic bias appears for strongly expansive transducers under large multiplicative noise, a case not often encountered in low-level vision. These results indicate that, for the transducer and noise models simulated here, MLDS recovers transducer shape. Our results illustrate how simulation can be used to evaluate and compare psychophysical methods by making their assumptions explicit.

## Introduction

How does our perceptual experience of the world relate to the physical world? This question is at the core of the study of perceptual psychology (Krueger, 1989). Mach (1865) proposed a principle of equivalence whereby “every psychical event corresponds to a physical event and vice versa.” In his Elements of Psychophysics, Fechner transformed this qualitative question and asked the quantitative question of how the magnitude of a person’s perceptual experience relates to the magnitude of a physical stimulus (Fechner, 1860). This marked the birth of the quantitative scientific study of perceptual psychology (Wichmann & Jäkel, 2018). Perceived magnitudes are private phenomena, and hence cannot be directly observed, but must be inferred from their expression in behavior. Fechner described three psychophysical methods to probe the relationship between perceptual and physical magnitudes: (1) the method of just noticeable differences, (2) the method of correct and incorrect cases, and (3) the method of mean errors. He conjectured that the three methods accomplish the same goal in complementary ways. Method (1) reveals the transition between noticeable and unnoticeable differences, method (2) counts noticeable differences, and method (3) addresses unnoticeable differences, but all three probe the same underlying perceptual representation. Fechner’s method (2) has become known as the method of constant stimuli, which has been used to measure perceptual sensitivity (i.e., just noticeable differences in discrimination or detection paradigms). His method (3) has become known as the method of adjustment, which has been used as a measure of appearance, that is, to measure suprathreshold perception.^1^ His method (1) has become known as the method of limits, referring to the procedure and sequence of stimulus presentation.

Interestingly, the methods that were supposed to measure the same internal representation of a physical stimulus took on a life of their own, resulting in different scientific approaches that have become difficult to reconcile. Starting in the 1950s and 1960s, there has been a divide between psychophysicists who measure sensitivity (e.g., Green & Swets, 1966) and psychophysicists who measure appearance (e.g., Stevens, 1957; Stevens, 1961). Measures of sensitivity probe the ability to detect small stimulus differences, rely on objective methods such as (m-alternative) forced-choice tasks, and quantify perceptual performance. Measures of appearance probe how stimulus attributes are perceived, rely on subjective methods such as ratings or adjustments, and quantify subjective experience (Kingdom & Prins, 2016). Each subfield has progressed largely independently up to a point where it became necessary to ask how the two measures are related and whether or not they are probing the same underlying dimension. These questions have been asked from a general theoretical point of view (e.g., Krueger, 1989; Norwich & Wong, 1997; Ross, 1997; Zhou, Duong, & Simoncelli, 2024), but also in individual stimulus domains, for example, concerning the relationship between apparent brightness and luminance detection thresholds (Heinemann, 1961; Maertens, Wichmann, & Shapley, 2015; Nachmias & Steinman, 1965; Whittle, 1992), color appearance and color discrimination (Hillis & Brainard, 2005; Hillis & Brainard, 2007b), or contrast sensitivity and contrast constancy (Georgeson & Sullivan, 1975; Peli, Arend, & Labianca, 1996).

One theoretical attempt to reconcile the two approaches is the concept of the transducer function (Wilson, 1980; Legge & Foley, 1980; Cannon & Fullenkamp, 1991), also known as transfer function (Georgeson, 1979), psychophysical law (Gescheider, 1997; Stevens, 1957; Treisman, 1964a), or true psychophysical function (Krueger, 1989). The transducer function is a hypothetical mapping that describes how a perceived magnitude (Ψ(*s*), e.g., brightness) varies as a function of changes in physical intensity (*s*, e.g., luminance) (Figure 1; Fechner, 1860). The internal axis of perceived magnitudes (Ψ(*s*)) cannot be directly observed but is probed in different ways by different tasks/paradigms. Sensitivity measures are thought to reflect the local slope (derivative) of the transducer function, while appearance measures are thought to reflect the transducer function itself. In either case, the hidden transducer function needs to be estimated from empirical data. However, empirical attempts to relate sensitivity and appearance measures via a transducer function have not always succeeded (e.g., Aguilar, Wichmann, & Maertens, 2017; Hillis & Brainard, 2007a; Li & Nagai, 2024; Maertens et al., 2015; Protonotarios, Johnston, & Griffin, 2016).

**Figure 1.**
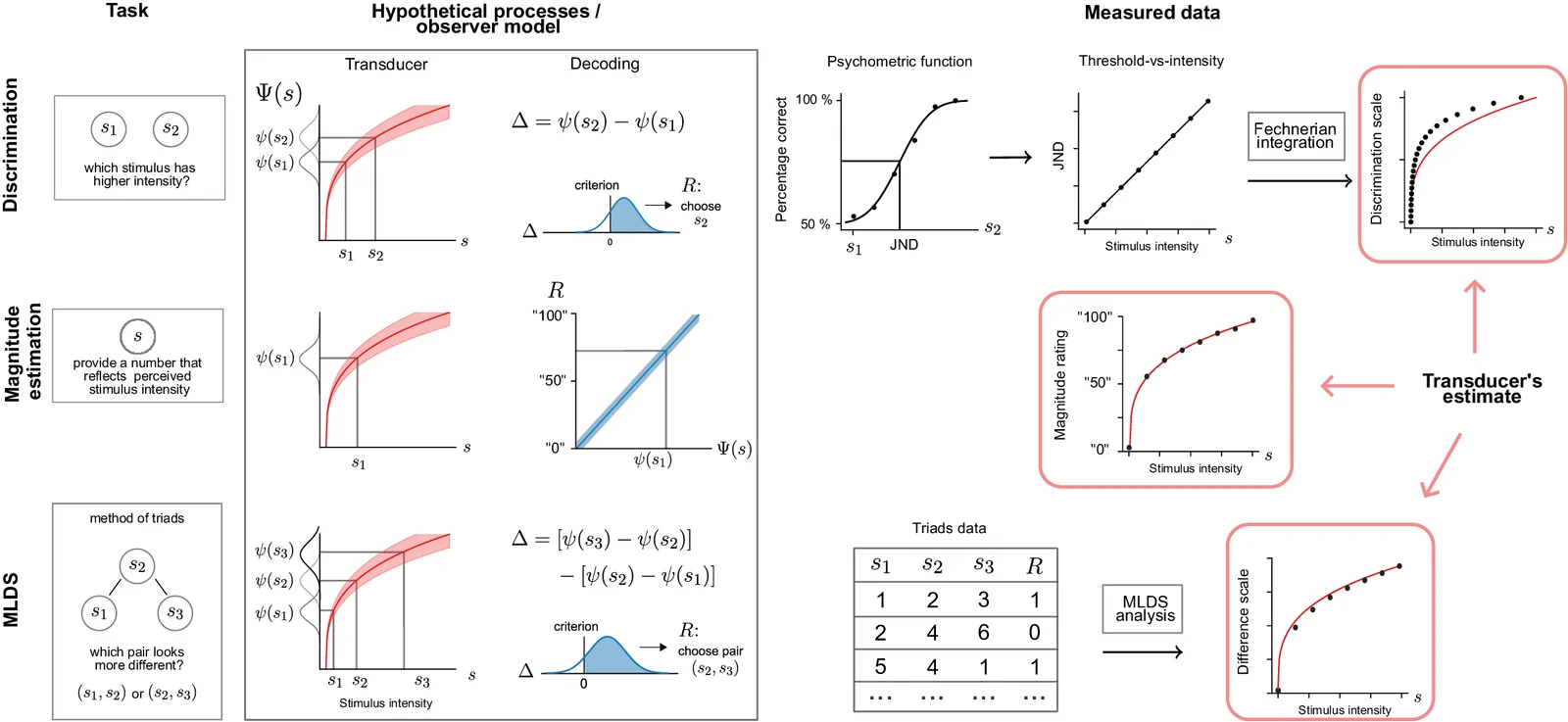
Experimental paradigms to probe observers’ transducer functions: discrimination, magnitude estimation, MLDS. Each paradigm uses a particular task. All paradigms assume a transducer (in red) but might differ with respect to assumptions about noise. Paradigms differ with respect to the “decoding” scheme applied by the observer (in blue). The data obtained with these paradigms can be used to derive the transducer. See text for details.

Maloney and Yang (2003) introduced a difference scaling procedure in which all stimuli are clearly visible. Observers judge three (or four) stimuli and report which of two pairs spans the larger perceptual difference, similar to Fechner’s method (2) but on a second-order comparison, a difference of differences. These suprathreshold comparisons are used in maximum likelihood estimation to derive a perceptual scale (maximum likelihood difference scaling, MLDS). Scales measured with MLDS have been used to estimate the transducer and to successfully predict threshold measurements (Cheeseman, Ferwerda, Maile, & Fleming, 2021; Devinck & Knoblauch, 2012; Knoblauch, Marsh-Armstrong, & Werner, 2020; Shi & Eskew, 2024; Shooner & Mullen, 2022). Zhou et al. (2024) proposed a formal approach to relate sensitivity and appearance measures using the statistical measure of Fisher information. They favor direct scaling methods to estimate the transducer and argue that suprathreshold scaling methods, such as MLDS, reflect integrated sensitivity and hence do not provide an independent estimate of the transducer function.

We argue that it is difficult to resolve the question of the relationship between appearance and sensitivity measures with an empirical strategy alone. We use numerical simulation to examine the assumptions involved in the various experimental approaches. In simulation, we adopt what we call a “God’s eye view,” because we decide about the transducer function, the noise model, and the decision rule (i.e., the components that are putatively involved in generating the data). We then test whether a certain method can “recover” the ground-truth transducer function from the generated data. In the following, we describe the hypothetical processes involved in classical sensitivity and appearance measurements. We state the assumptions and procedural steps involved in each type of measurement and discuss their respective benefits and limitations. In the second part, we describe our simulation strategy and present our results.

## Paradigms that estimate the transducer

### Discrimination experiments

Sensitivity or discriminability characterizes visual performance at its limits. It is measured under conditions where stimuli are barely visible or differences between stimuli are tiny. Observers are presented with two (or more) stimuli and indicate with a button press whether a stimulus was present or which of the two stimuli was larger (*A* > *B*). Stimuli vary along some dimension of interest (*s*; e.g., contrast, spatial frequency; Figure 1A), and performance is measured at many different stimulus levels. Visual sensitivity is quantified in terms of a threshold or just noticeable difference (JND).

To predict the binary response from the internal stimulus representation (Ψ(*s*)), an explicit decision rule (also called readout, decoding) is formulated. It is based on the perceived difference (Δ) between the two stimulus values in each trial (Ψ(*s*_1_) and Ψ(*s*_2_); see Figure 1, top row). With a fixed transducer function, the same stimulus elicits the same percept every time it is presented, and an observer would give the same response. This is not what is observed in psychophysical experiments where responses vary from trial to trial. To account for this variability, the transducer function is modeled with “noise” in the perceptual dimension (Figure 1, distribution of values on the y-axis of the transducer) (e.g., Georgeson & Meese, 2006; Kingdom, 2016). When the difference between the to-be-compared stimuli is small, the noise can have a considerable effect on observers’ responses. In the presence of noise, the response is derived by comparing the difference, Δ, to a criterion (e.g., in signal detection theory; Figure 1, upper panels, in blue).

In practice, we neither know the transducer nor the noise but need to derive them empirically. In discrimination experiments, this is done via Fechnerian integration (Baird, 1978; Gescheider, 1997), which requires extensive data collection. The transducer is estimated from JNDs, which need to be measured at different levels of stimulus intensity. Each such JND measurement is derived from a psychometric function, which, in order to provide a reliable estimate of discrimination performance, involves 100 to 150 trials depending on the number of stimulus levels (see, e.g., Wichmann & Jäkel, 2018). To be able to meaningfully integrate JNDs, it is assumed that each JND evokes an equal increase in perceived magnitude in the internal stimulus representation (Ψ(*s*); Laming, 1997). Starting at the lowest JND (at detection threshold), JNDs are cumulatively summed, keeping equidistant steps on the scale value and assigning the corresponding physical stimulus differences on the x-axis. This procedure, more informally known as “summing JNDs,” constructs a scale from discriminability data alone (Figure 1).

However, it is not guaranteed that a JND reflects equal distance along the internal dimension. JNDs are defined in empirical terms (e.g., the point at which the observer correctly responds 75% of the time for a two-alternative forced choice task), and we have no independent means to probe the internal distance other than by evaluating performance. If the noise increases with stimulus magnitude (multiplicative noise), this will produce increasing JNDs for a linear transducer. The same linear transducer with additive noise (constant noise magnitude, irrespective of stimulus magnitude) will produce JNDs of fixed size. Thus, JNDs reflect distances along perceived magnitude only relative to the effect of noise.

In Figure 2, we illustrate this ambiguity with a compressive and a linear transducer, each with multiplicative noise. For the linear transducer, noise magnitude increases more rapidly with stimulus intensity than for the compressive transducer (noise magnitude 0.1 vs. 0.33, reflected on the shaded red areas in Figure 2). Data generated from these two transducer scenarios produce identical JNDs, because as long as the noise magnitude and the nonlinearity remain in a constant relationship, JNDs will be identical.

**Figure 2.**
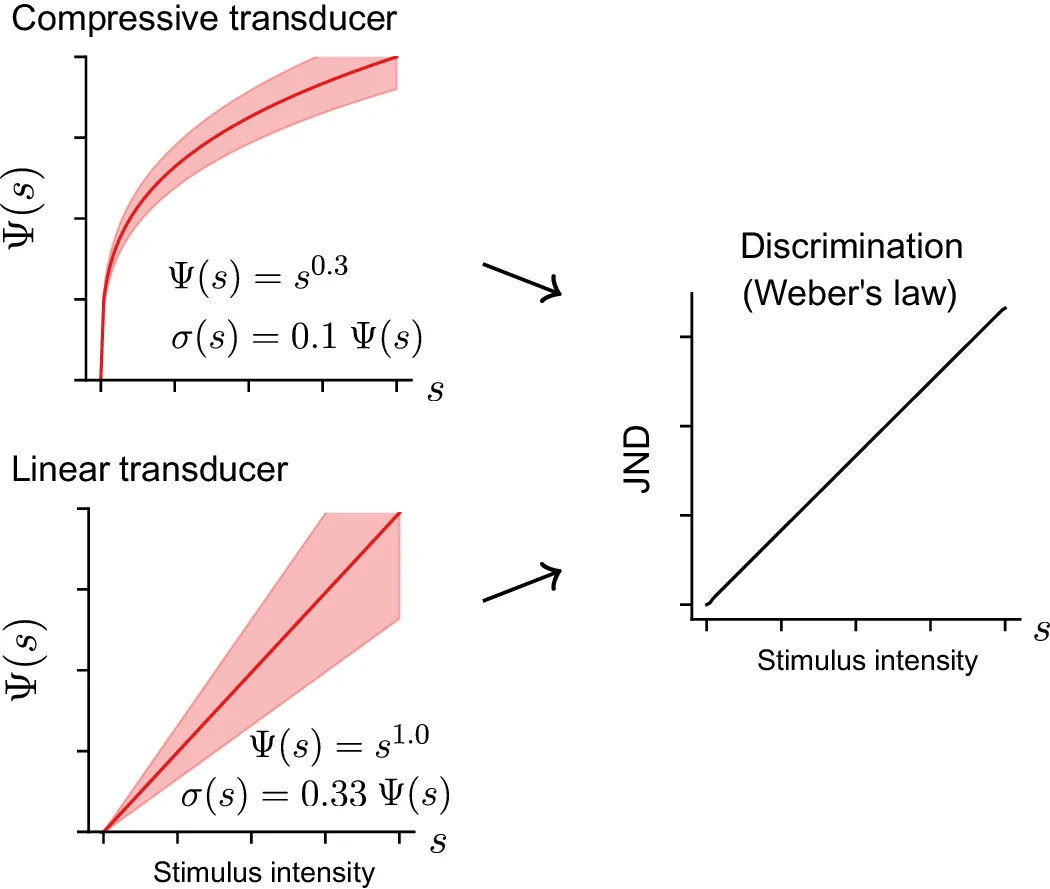
Limitations of discrimination experiments. Both the shape of the transducer and its noise determine discriminability (JNDs). A compressive transducer (*top*) produces identical JNDs to a linear transducer (*bottom*), both under multiplicative noise. A scale derived from these data will also be identical and, in this case, compressive, not necessarily reflecting the underlying transducer. Adapted from Zhou et al. (2024).

Thus, data from a standard two-alternative forced-choice discrimination experiment is ambiguous and cannot be used to determine the transducer’s shape independent of the noise. Alternative paradigms have been developed that tackle this problem, for example, by introducing a second response (Solomon, 2007) (how to disentangle noise and transducer empirically from discrimination data is a research agenda in its own right; e.g., Georgeson & Meese, 2006; Klein, 2006; Kontsevich, Chen, & Tyler, 2002; Malo, Esteve-Taboada, Aguilar, Maertens, & Wichmann, 2025).

### Direct methods

Direct experimental procedures, such as matching and scaling, measure the appearance of a stimulus (i.e., how it looks) in a direct way. Stimuli are clearly visible (above threshold), and observers compare them to numerical references (scaling) or to references in the stimulus domain (matching). In direct scaling, specifically with the method of magnitude estimation (ME; Stevens, 1960; Stevens, 1975), observers assign a numerical value to each perceived stimulus intensity (Figure 1, middle row). In such suprathreshold experiments, the trial-to-trial variability has a relatively smaller effect on individual estimates. Therefore, only a few repeats are necessary to estimate the perceived intensity at one stimulus level (usually three to five; Marks & Gescheider, 2002).

To get from the internal stimulus representation (Ψ(*s*)) to a response (i.e., the readout), the mapping between perceived magnitudes and the assigned numerical values (i.e., the internal “number line”) is assumed to be linear (Figure 1, middle row). This assumption is not uncontroversial, and nonlinearities in the mapping have been documented and discussed extensively in the literature (see, e.g., Gescheider, 1997; Krueger, 1989; Shepard, 1981; Treisman, 1964b). Figure 3 illustrates the ambiguity in magnitude estimation. A compressive and an expansive transducer are combined with two different “mappings” to the number line, a linear and a compressive one. Both cases result in identical scales. Thus, despite their appeal to be a straightforward measure, direct scales do not guarantee to reflect the underlying transducer either. Matching procedures suffer from similar ambiguities (see Vincent, Maertens, & Aguilar, 2024).

**Figure 3.**
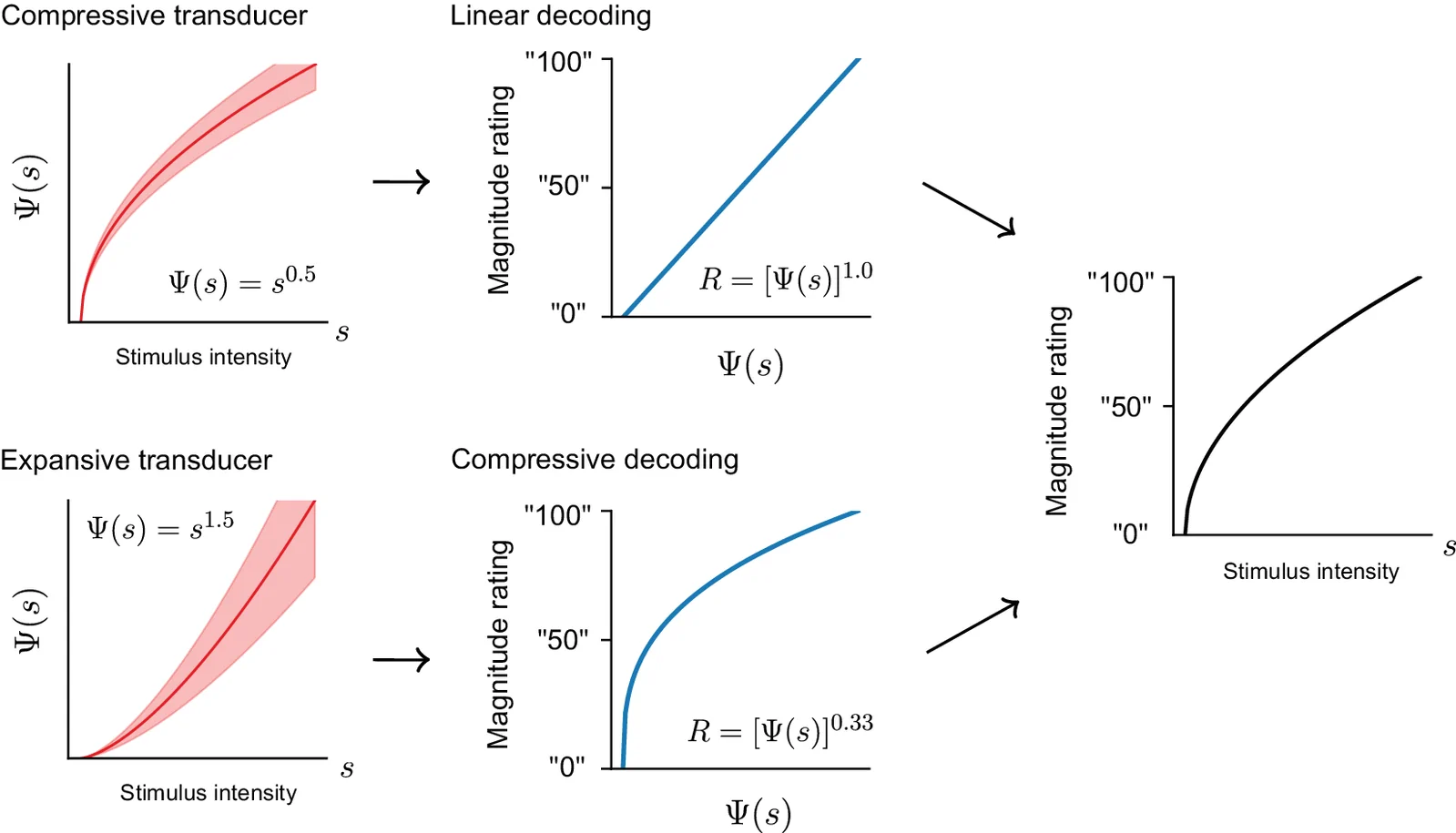
Limitations of magnitude estimation experiments. A magnitude estimation experiment requires assuming that the mapping between perception Ψ(*s*) and the number line is linear. Identical outcome (*right*) can be produced by different combinations of transducers (*left*) and number line mappings (*middle*).

### Difference scaling methods

In difference scaling methods, observers judge differences between stimuli that are clearly visible. The first experiment of this type is attributed to Plateau (1872), who asked observers to bisect a range of grays into two perceptually equal intervals (Laming & Laming, 1996). More modern approaches, such as MLDS (Knoblauch & Maloney, 2012; Maloney & Yang, 2003), combine aspects of discrimination and scaling. MLDS collects data with the method of triads (or quadruples), that is, three (or four) stimuli are presented, and the observer judges which of the stimulus pairs comprises the larger difference, in a forced-choice manner (Figure 1, bottom panels). This results in binary data and therefore requires several repeats per comparison to increase the precision of the estimated scale.

Measurement theory has established that difference judgments can be used to construct an interval scale (Krantz, Luce, Suppes, & Tversky, 1971) but did not include a model of noise. MLDS assumes a noise model that makes it possible to determine a scale from binary data. The assumed noise is Gaussian of constant variance across the transducer values. For triads, three stimuli are presented in each trial and comprise two perceptual differences, the intervals [Ψ(*s*_3_) − Ψ(*s*_2_)] and [Ψ(*s*_2_) − Ψ(*s*_1_)]. The observer decides which of the intervals is larger. This is expressed in the following decision rule:

(1) $$\begin{array}{c} \Delta =\left[\Psi \left(s_{3}\right)-\Psi \left(s_{2}\right)\right]-\left[\Psi \left(s_{2}\right)-\Psi \left(s_{1}\right)\right]+\varepsilon ,\; \end{array}$$

where Δ is the decision variable. When Δ > 0, the observer perceived the difference between *s*_2_ and *s*_3_ to be larger than for *s*_1_ and *s*_2_. The decision variable is assumed to be corrupted by Gaussian noise of unknown variance (ϵ ∼ *N*(0, σ^2^)), which models the stochasticity observed in behavioral data. In this model, the transducer values (Ψ(*s*_*i*_)) and the variance of the noise (σ) are free parameters that are estimated from the data. Finding the parameters (i.e., the scale values) is done via maximum likelihood estimation, and an implementation of this is available in the R programming language (Knoblauch & Maloney, 2012).

When introducing the method, Maloney and Yang (2003) extensively tested in simulation that MLDS recovers the transducer when noise is Gaussian. They also showed this with multiplicative noise, but only for a single noise magnitude. Here we test the robustness of MLDS with multiplicative noise systematically across a broad range of transducer shapes and noise magnitudes. The theoretical reason why difference scaling recovers the transducer for various underlying noise distributions was outlined by Kingdom (2016). As the name says, observers report a difference of perceptual magnitudes or, as in MLDS, a difference of differences (Equation 1). The expected value of a difference of two independent random variables equals the difference of their means. The variance of the difference distribution equals the sum of the variances of the original distributions. Consequently, the expected value of the difference-of-differences decision variable in MLDS is determined solely by the shape of the transducer, irrespective of the noise magnitude (Kingdom, 2016).

### Interim summary

We have presented and discussed the hypothetical processes involved in discrimination, direct scaling, and difference scaling. Standard 2-AFC discrimination data conflate the shape of the transducer with its noise (Zhou et al., 2024), so that it cannot be used alone to estimate the transducer and requires additional assumptions. Data from direct scaling methods, such as magnitude estimation, conflate the transducer’s shape with the function that maps perceptual magnitude to numbers. MLDS seems to offer an alternative to circumvent these problems, as it explicitly states the model about what observers do.

In the following, we simulate MLDS experiments from known ground-truth transducer functions of different shape and with different types of noise. In each simulation, we generate synthetic data from one ground-truth transducer and use MLDS to estimate a perceptual scale for each dataset. The estimated scale is compared to the ground-truth scale to evaluate how well MLDS can recover it.

## Simulations

We simulate MLDS experiments for different transducer and noise combinations. We tested one logarithmic and seven power-law transducers together with additive (4 levels) and multiplicative noise (16 levels). We additionally simulated Poisson noise, as a special asymmetric case of variable noise; see Supplementary Material for details. We simulated all combinations of these parameters. There is a subset of combinations for which sensitivity is identical. This is the critical test to see whether MLDS can recover the underlying transducer’s shape. The other parameter combinations produced data with different sensitivity, for which we evaluate how well MLDS recovers the transducer shape for different noise magnitudes.

### Transducers and noise

A transducer function specifies how a stimulus intensity (*s*) is mapped into a noisy perceptual representation (ψ(*s*)). We model this perceptual representation as a Gaussian random variable,

(2) $$\begin{array}{c} \psi \left(s\right)∼N(\mu \left(s\right),\sigma^{2}\left(s\right)).\; \end{array}$$

μ(*s*) is the function that defines how the transducer’s mean relates to stimulus intensity *s* (i.e., its shape), and σ^2^(*s*) is the function that defines how noise magnitude relates to stimulus intensity. From the universe of possible transducer function shapes, we chose those that can produce identical Weber’s law sensitivity for different noise types (Figure 4). The first choice is a logarithmic transducer with fixed additive noise (i.e., noise magnitude is independent of stimulus intensity). This choice of transducer originates with Fechner (1860), as the integration of Weber’s law sensitivity. Identical Weber’s law sensitivity can result from a power-law transducer with multiplicative noise (i.e., noise magnitude increases with stimulus intensity). This is our second choice of transducer, additionally motivated by a line of research that originates with magnitude estimation (Stevens, 1957; Stevens, 1960), which has argued that the transducer function is a power law across various stimulus domains (Stevens, 1975).

**Figure 4.**
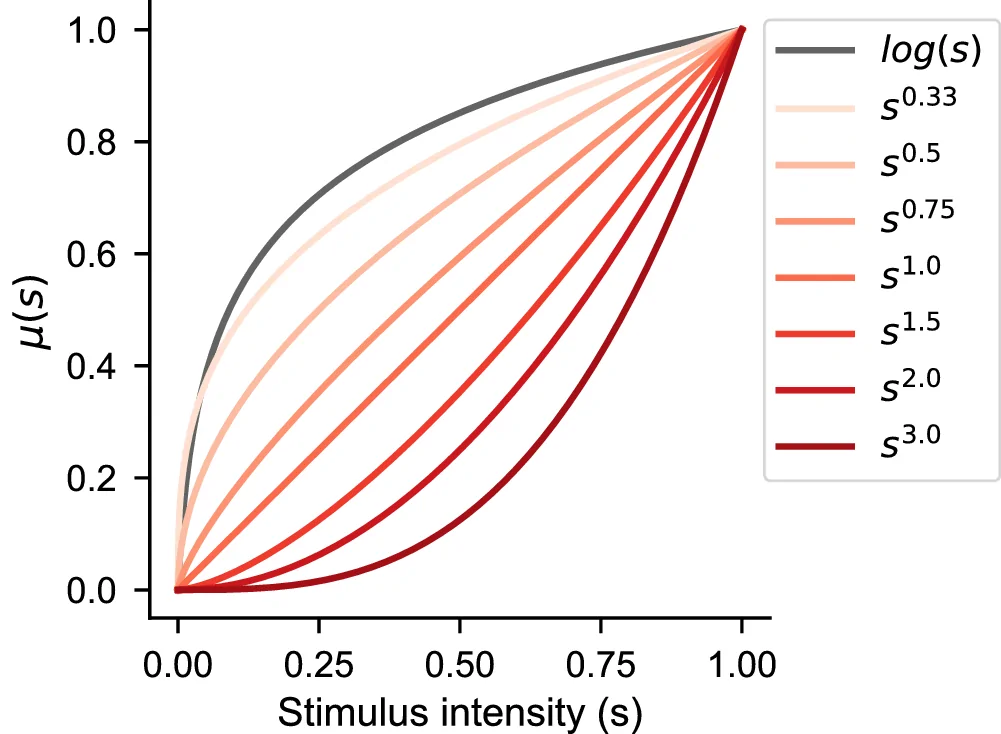
Simulated transducer functions.

The power-law transducers were defined as

(3) $$\begin{array}{c} \mu \left(s\right)=s^{\alpha },\; \end{array}$$

where μ(*s*) is the mean of the transducer, *s* the stimulus intensity, and α the exponent. We simulated seven different α values (0.33, 0.5, 0.75, 1.0, 1.5, 2.0, 3.0). Values smaller than 1 correspond to a compressive transducer and values greater than 1 to an expansive transducer.

The logarithmic transducer was defined as

(4) $$\begin{array}{c} \mu (s)=\log s,\; \end{array}$$

with μ(*s*) being the mean of the transducer and *s* stimulus intensity. As the logarithmic function is positive only for values greater than 1, stimulus intensities were chosen to vary between 1 and 100. However, to visualize both transducer types with a common x-axis, we rescaled the stimulus intensities later to the range 0 to 1. Additionally, to keep the noise magnitude interpretable relative to the range of the transducer, we normalized μ(*s*) for this transducer to the range 0 to 1, to match the range of the power-law transducers. This normalization affected the mean of the transducer μ(*s*), but it did not restrict the values of the simulated (noisy) perceptual representation ψ(*s*) (Equation 2); the latter were not clipped to any range.

Additive noise was independent of stimulus intensity.

(5) $$\begin{array}{c} \sigma (s)=\sigma ,\; \end{array}$$

Multiplicative noise grows with stimulus magnitude at a rate controlled by parameter *g*:

(6) $$\begin{array}{c} \sigma (s)=g\mu (s).\; \end{array}$$

#### Calculation of noise magnitude

As explained in the previous section, data from discrimination and magnitude estimation experiments do not allow the unambiguous inference about transducer shape and noise. To test in simulation whether MLDS experiments could offer a remedy from this problem, here we purposefully create data that are identical in sensitivity, but with different underlying transducer–noise combinations. To do so, we specify our data to have the following four Weber fractions *k*: 5%, 7.5%, 10%, and 20% that are commonly encountered in different perceptual domains (Baird, 1978).

For the logarithmic transducer function and additive noise, those Weber fractions are produced when the noise magnitude σ is equal to the Weber fraction itself. Thus, in the simulations, we used σ of 0.05, 0.075, 0.1, and 0.2.

For the power-law transducer, the Weber fraction *k* is determined by

(7) $$\begin{array}{c} k=\frac{g}{\alpha }\; \end{array}$$

(see Appendix A for derivation). We calculated the *g* values necessary to produce the specified Weber fractions (0.05, 0.075, 0.1, and 0.2) for all seven exponents α. This resulted in 16 unique *g* values, which we tested in simulation (see Appendix B for the full list).

### Simulation

#### Experimental design and observer model

We simulate the method of triads, where an observer is presented with three stimuli, *s*_1_, *s*_2_, *s*_3_, and decides which pair, (*s*_1_, *s*_2_) or (*s*_2_, *s*_3_), has the greater perceived difference. We used 10 stimulus intensities (*s*) linearly spaced from 0 to 1. This allows for 120 possible combinations of nonoverlapping triads (*s*_1_ < *s*_2_ < *s*_3_ or *s*_1_ > *s*_2_ > *s*_3_). We repeated each 10 times, resulting in a total of 1,200 trials per simulation.

On each trial, the three stimulus intensities of a triad are mapped to an internal representation through one of the transducers described above. Each of these representations is subjected to the specified noise (see Equation 2). Then the simulated observer calculates a decision variable with these noisy representations:

(8) $$\begin{array}{c} \Delta =\left[\psi \left(s_{3}\right)-\psi \left(s_{2}\right)\right]-\left[\psi \left(s_{2}\right)-\psi \left(s_{1}\right)\right],\; \end{array}$$

and assigns a binary response according to the following decision rule:

(9) $$\begin{array}{c} R=\left\{\begin{array}{c} (s_{2},s_{3})\;\text{if}\;\Delta >0\\ (s_{1},s_{2})\;\text{otherwise}. \end{array}\right.\; \end{array}$$

Note that we assume a noiseless decision (compare Equations 1 and 8); all variability in the responses is due to the noise present in the transducer. The simulated data contain all triads (*s*_1_, *s*_2_, *s*_3_), and the responses *R* are added and fed into the MLDS routine for scale estimation.

#### Scale estimation with MLDS

We use the MLDS implementation by Knoblauch and Maloney (2012) wrapped for Python (Aguilar, 2025). In short, the estimation routine finds scale values for each stimulus intensity by maximizing the likelihood of the input data (for details on MLDS, see Aguilar et al., 2017; Maloney & Knoblauch, 2020; Wiebel, Aguilar, & Maertens, 2017). The optimization is framed as a generalized linear model, in which the response to each triad is modeled as a Bernoulli variable, and the regression coefficients are the scale values. The output of MLDS is an interval scale, that is, invariant to linear transformation (scaling and shift). As such, its zero point is arbitrary and is by default set to the first stimulus intensity. MLDS also estimates a noise parameter, which captures the observer’s stochasticity. In the default parametrization, the scale’s maximum is the inverse of the noise magnitude; hence, the higher the scale maximum, the lower the noise. Here, we used an alternative parametrization in which the maximum is fixed at 1 and the noise parameter is given separately as a fitted parameter. This parametrization facilitates the separate evaluation of the shape of the scale and the associated noise.

One should be aware that the estimated noise parameter is different from the noise σ(*s*) that we inject at the perceptual representation of each stimulus (Equation 2). In our simulations, the decision is noiseless, so noise enters only at the stimulus representation, from where it propagates to the decision variable (the difference of differences, Equation 8). For a triad, the difference is Δ = ψ(*s*_1_) − 2ψ(*s*_2_) + ψ(*s*_3_), and its variance is Var(Δ) = σ^2^(*s*_1_) + 4σ^2^(*s*_2_) + σ^2^(*s*_3_). With additive noise, this reduces to 6σ^2^ (for quadruples, 4σ^2^). The estimated noise parameter is thus a fixed multiple of σ (here, $\sqrt{6}\;\sigma$). With multiplicative noise, the transducer noise σ(*s*) grows with stimulus intensity, and consequently, the variance of Δ depends on the transducer shape. A single noise parameter cannot capture this dependence, so its relationship to σ(*s*) is not identifiable.

#### Evaluation

To assess whether MLDS provides good and unbiased estimators of the ground-truth transducers, we compute the difference between estimated $\hat{\Psi }\left(s_{i}\right)$ and ground-truth transducer values,

(10) $$\begin{array}{c} e_{i}=\hat{\Psi }\left(s_{i}\right)-\mu \left(s_{i}\right),\; \end{array}$$

where $\hat{\Psi }\left(s_{i}\right)$ is the scale value for the *i*th stimulus *s*_*i*_ and μ(*s*_*i*_) the transducer value for that same stimulus. For each simulation, we use these differences to quantify the goodness of the estimation as the root mean squared error (RMSE):

(11) $$\begin{array}{c} RMSE=\sqrt{\frac{1}{n}\sum_{i=1}^{n}e_{i}^{2}}\; \end{array}$$

where *e*_*i*_ is the error defined above and *n* the total number of stimuli (*n* = 10). We analyze the distribution of errors, *e*_*i*_, to check for potential bias in the MLDS estimation (see Supplementary Material for details).

To test the ability of MLDS to recover the parameters of the ground-truth transducer, we fit each simulated perceptual scale with a power-law function (Equation 3) and compared the fitted parameters to the ground truth. We repeat this procedure for all transducer and noise combinations and compare the distribution of parameters across simulations against the ground-truth parameter value. The fitting was done with a least squares criterion.

All simulations were implemented in Python (3.12), using the NumPy, SciPy, pandas, Matplotlib, and seaborn scientific computing libraries. Scale estimation with MLDS additionally used R (4.5) and the MLDS R package (0.5.1). All code and data are available at https://github.com/computational-psychology/aguilar_maertens_vincent_2026.

### Results

#### Scale estimation with additive and multiplicative noise

Figures 5 and 6 show the simulation results for transducers with additive and multiplicative noise, respectively. In most cases, the estimated scale values (black) line up with the ground-truth transducer functions (red).

**Figure 5.**
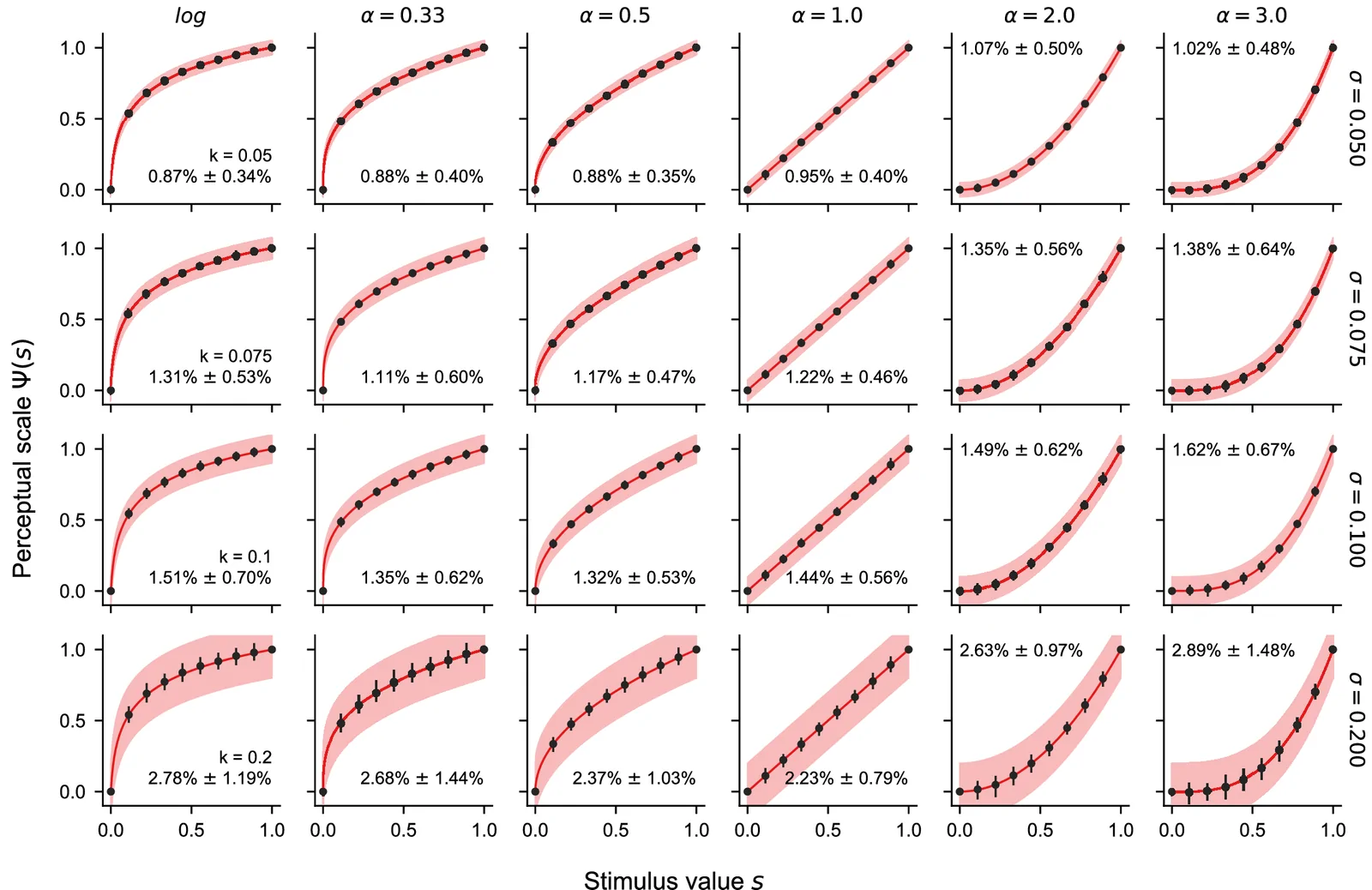
Simulation results for transducers with additive noise. Logarithmic and power-law transducers of varying exponent (columns) were used to simulate comparative judgments in an MLDS experiment, with varying amounts of additive noise (σ, rows). For the logarithmic transducer, the expected Weber fraction *k* is indicated in each panel. Black circles and error bars depict the mean and 95% confidence intervals of the estimated scales across 100 simulations. The ground-truth mean of the transducer is depicted as a continuous red line, its noise magnitude as the red area (1 *SD*). The red area is truncated at the upper edge of each panel for display only; the underlying noise distributions were not restricted to any range (see Methods). Deviation from ground truth was quantified as RMSE, shown as percentages inside each panel (mean ± *SD*).

**Figure 6.**
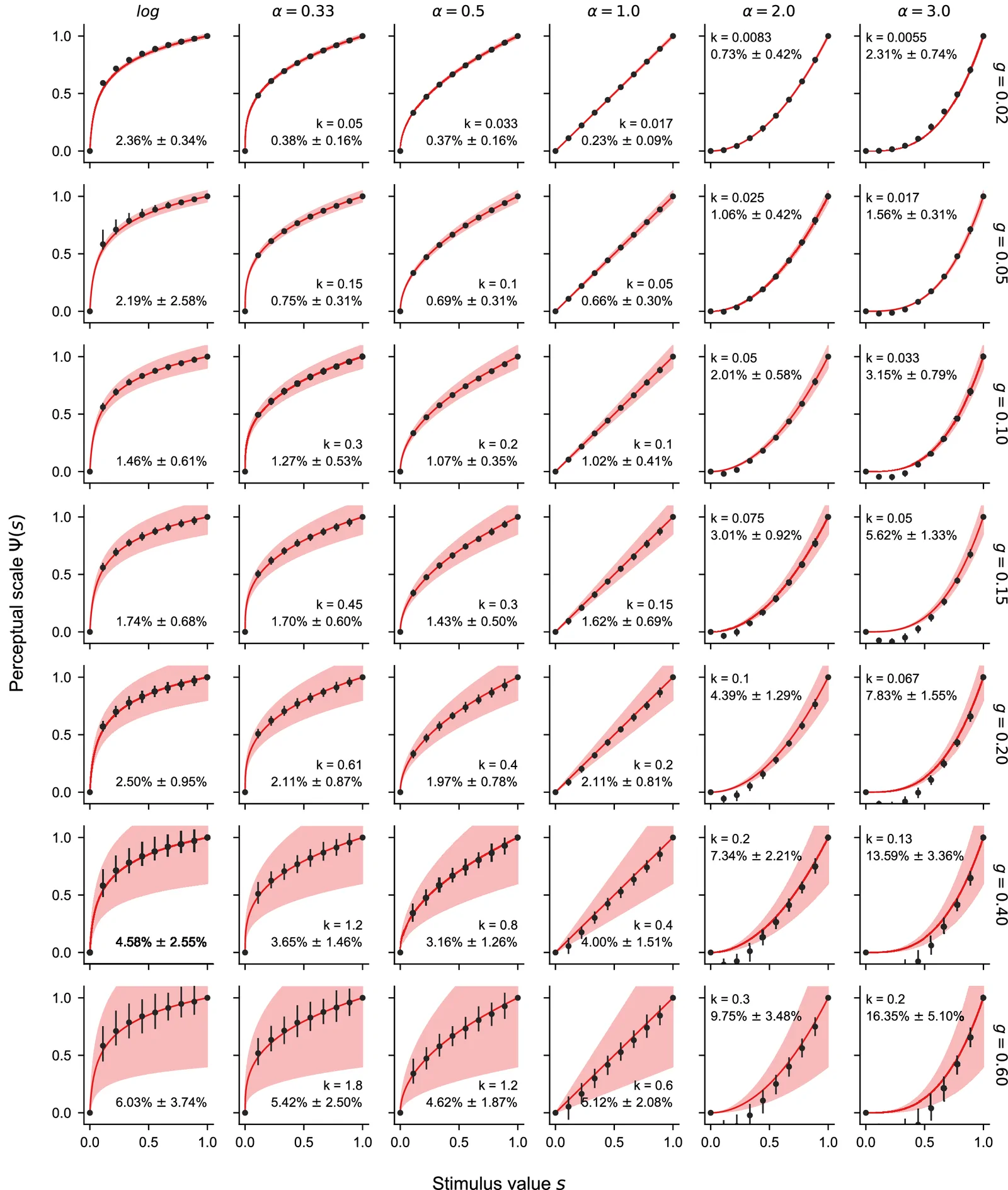
Simulation results for transducers with multiplicative noise. Logarithmic and power-law transducers of varying exponent (columns) were used to simulate comparative judgments in an MLDS experiment, with varying amounts of multiplicative noise (*g*, rows). The expected Weber fraction *k* is indicated in each panel. Black circles and error bars depict the mean and 95% confidence intervals of the estimated scales across 100 simulations. The ground truth mean of the transducer is depicted as a continuous red line, its noise magnitude as the red area (1 *SD*). The red area is truncated at the upper edge of each panel for display only; the underlying noise distributions were not restricted to any range (see Methods). Deviation from ground truth was quantified as RMSE, shown as percentages inside each panel (mean ± *SD*). Only a subset of possible *g* values is shown here for clarity.

Deviations occur for expansive transducers and with higher multiplicative noise (*g* > 0.10 and α ⩾ 2, two rightmost columns in Figure 6). We quantify the deviation from ground truth with the average error (RMSE) for each simulation (Equation 11). For both additive and multiplicative noise, RMSE increased with increasing simulated noise magnitude. For logarithmic and power-law transducers with additive noise (Figure 7A), RMSE ranged from 0.2% to 2.7% at the lowest noise magnitude (σ = 0.0165) and from 4.3% to 5.4% at σ = 0.4, the highest magnitude shown. For compressive and linear transducers (α ⩽ 1) with multiplicative noise (Figure 6), RMSE ranged from 0.2% to 2.4% for the lowest noise magnitude (*g* = 0.0165) and from 4.6% to 6.0% for the highest noise magnitude (*g* = 0.6). For expansive transducers (α ⩾ 2.0) with multiplicative noise (Figure 6, two rightmost columns), RMSE ranged from 0.7% to 2.4% for the lowest noise magnitude (*g* = 0.0165) and ranged from 9.8% (α = 2) to 16.4% (α = 3) for the highest noise magnitude (*g* = 0.6).

**Figure 7.**
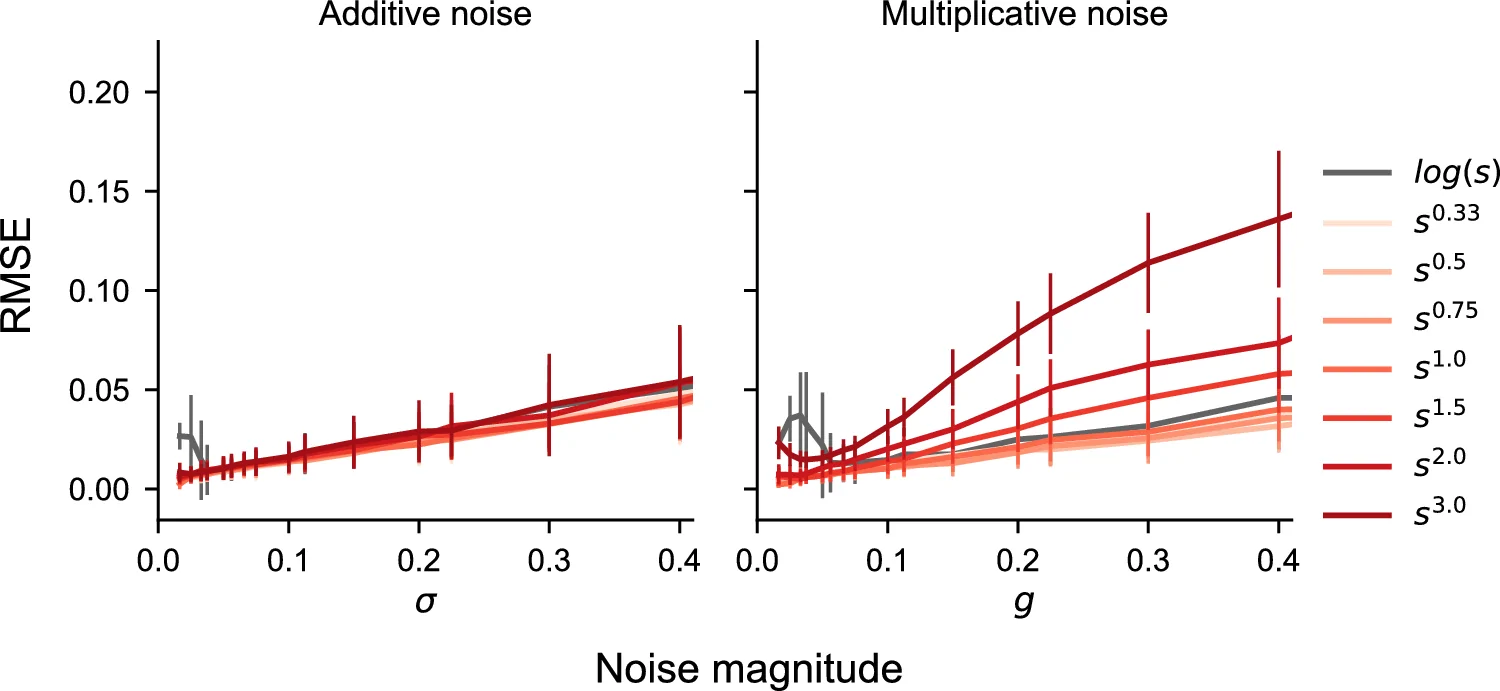
RMSE between ground truth and scales estimated with MLDS, for transducers with additive (left) and multiplicative (right) noise.

We also explored the distribution of errors as a function of stimulus intensity to check for potential bias in the estimation. For all but the expansive transducers, errors were uniformly distributed across different stimulus levels, showing no indication of systematic bias (see Supplementary Material). For the expansive transducers, MLDS systematically underestimates the scale values at low intensities, resulting in skewed error distributions (see Supplementary Material for details).

The same overall pattern of results was found with Poisson noise, as a special case of variable noise (see Supplementary Material for details).

To visually compare the goodness of transducer estimation, we plot the RMSE in all simulated scenarios of transducer function shape, noise types, and noise magnitudes (Figure 7). For transducers with additive noise, RMSE increases linearly with noise magnitude and with similar slope and intercept across different transducer shapes (slopes 0.10–0.12 and null intercept). For compressive or linear transducers with multiplicative noise, RMSE also increases roughly linearly with noise magnitude (slopes 0.07–0.09). For the most expansive transducer (α = 3), the slope was 0.3.

#### MLDS can estimate the transducer for cases of identical sensitivity

The different panels in Figure 8 show subsets of ground-truth transducer functions, which, in combination with different types of noise, produce equal discriminability in simulation. Equal discriminability was defined as equal Weber fractions (*k*). We used a logarithmic transducer with additive noise (in gray) and various power-law transducers with multiplicative noise (in orange and red). Ground-truth functions are depicted by continuous lines and estimated scale values by points. MLDS recovers the shape of the simulated transducers for different noise types in most cases, except for the expansive transducers (α ⩾ 2). Here, the estimated scales underestimate the ground-truth scale at low- to mid-stimulus intensities (as shown in Figure 6), that is, across the range of the scale where physically different stimuli all map onto the same perceived value. This “dip” is bigger for larger Weber fractions, and more pronounced, the more expansive the transducer. It is present for α = 2 at all four Weber fractions and largest for α = 3. The scales’ variability was also larger for larger Weber fractions, which is expected because the simulated noise is also larger.

**Figure 8.**
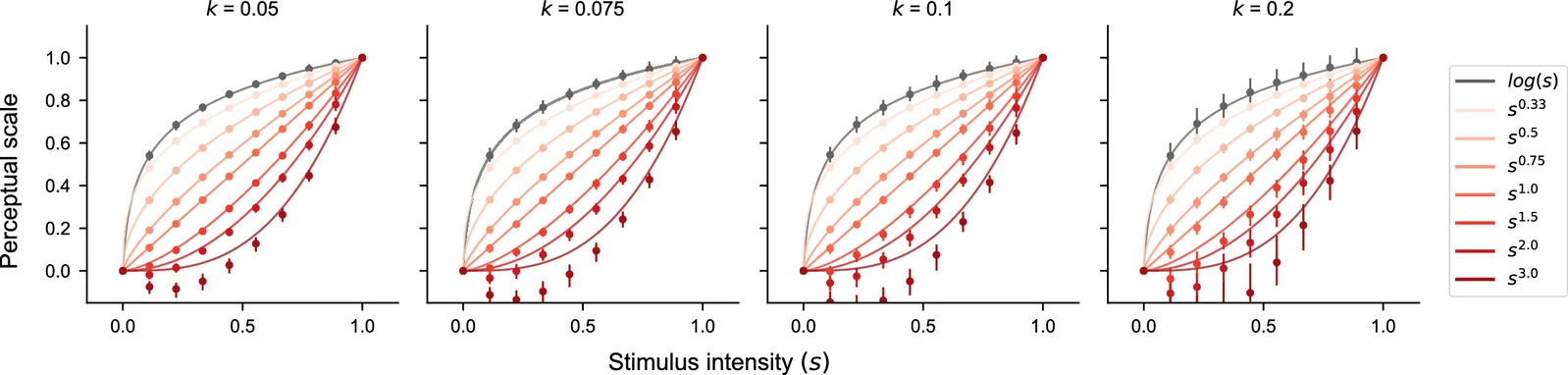
Simulation results for transducers that produce identical discriminability. The logarithmic transducer had additive noise (gray), while the power-law transducers had multiplicative noise (orange to red). In each panel, all transducers are expected to produce the same Weber fraction (*k*), as indicated in the respective panel titles. Lines depict the ground-truth transducer mean; colored circles and error bars depict the mean and 95% confidence intervals of the estimated scales across 100 simulations.

#### Recovery of power-law exponent by MLDS

To quantify how well MLDS recovers the transducer shape, we fitted the exponent of a power-law function for each simulated scale. Figure 9 shows the distribution of the estimated exponents $\hat{\alpha }$ for all simulations. For additive noise, the estimated exponent coincides with the ground-truth exponent, and the spread of the parameter increases with increasing noise magnitude (σ).

**Figure 9.**
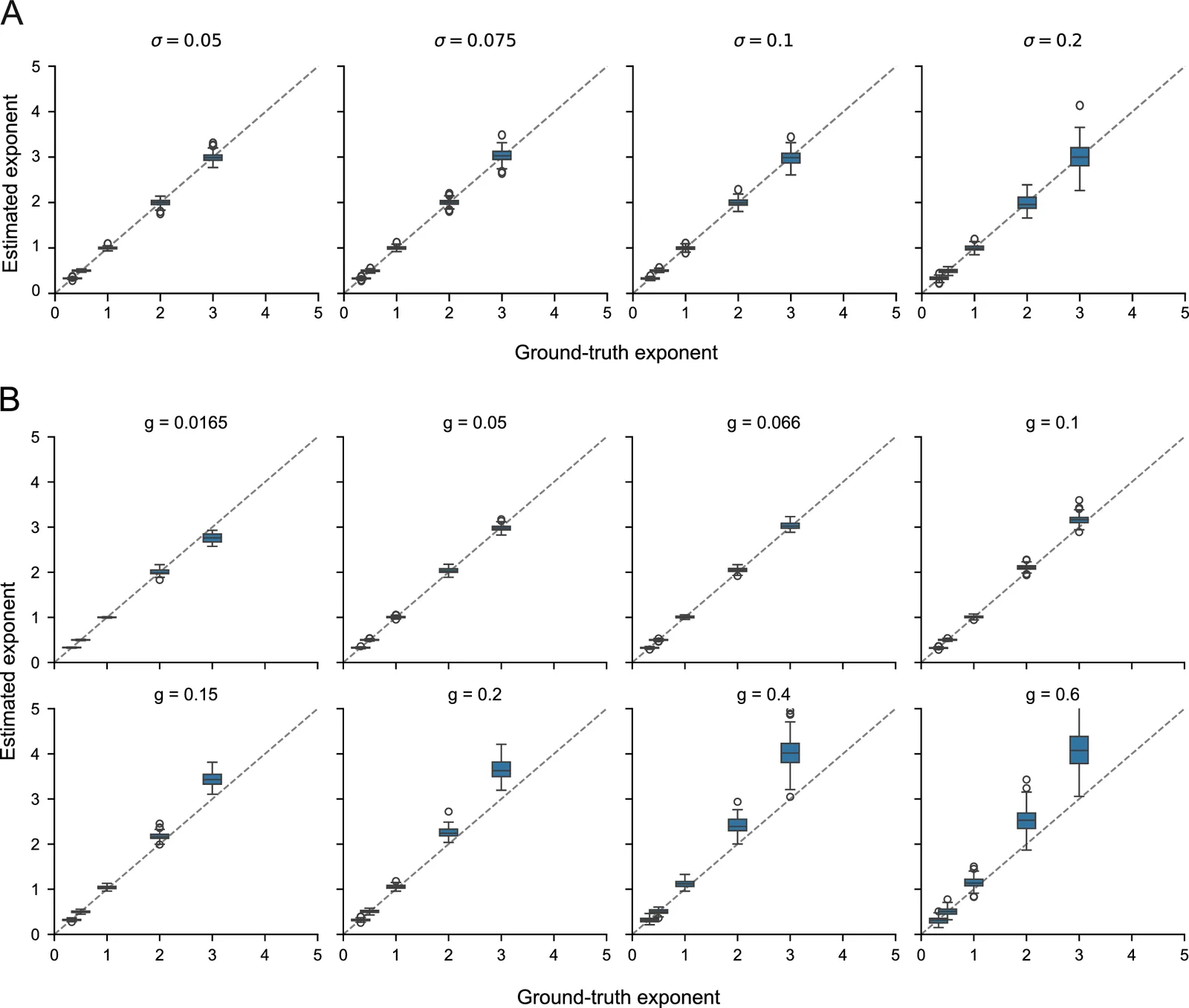
Recovery of the transducer exponent α from MLDS scales. The estimated exponents (y-axis) were obtained by fitting each simulated scale to a power-law function. The boxplots show the distribution of the estimates across 100 simulations, run for different transducer shapes (x-axis), levels of noise (σ or *g*, panels), and noise type, additive (**A**) and multiplicative (**B**). The diagonal dashed line indicates the identity line. Only a subset of possible *g* values is shown here for clarity.

For multiplicative noise, the estimated exponent coincides with the ground-truth exponent for lower noise magnitudes (*g* less than 0.1) and compressive or linear transducers (α ⩽ 1). For the expansive transducers, the estimated exponents deviate from the ground-truth exponents as noise magnitude increases (Figure 9B).

## Discussion

We provided theoretical arguments why data from discrimination and direct scaling experiments do not sufficiently constrain estimates of the so-called transducer function (i.e., the mapping between physical and perceived stimulus magnitude). Using simulation, we tested whether MLDS has the capacity to estimate the known ground-truth transducer function from data generated with different transducer and noise types. For the scenarios tested here (i.e., power-law and logarithmic transducer function shapes), MLDS recovered the underlying transducer well when noise was additive, irrespective of noise magnitude. When noise was multiplicative, MLDS still recovered compressive and linear transducer shapes well. For expansive transducer functions and larger noise magnitudes, we observed systematic bias in function estimation. Extending previous work by Maloney and Yang (2003) and Kingdom (2016), our simulations establish that MLDS is robust to multiplicative noise across a wide range of transducer shapes and noise magnitudes.

We detailed in the section “Paradigms that estimate the transducer” that transducer shape and noise cannot be uniquely determined from discriminability (sensitivity) data alone. To specifically test the ability of MLDS to disentangle transducer shape and noise, we included cases in the simulation where discriminability was identical for different transducer and noise combinations. The scales estimated with MLDS in these cases recovered the known ground-truth transducer shape. Hence, we conclude that MLDS can disambiguate transducer shape and noise for the range of transducer and noise models we simulated. MLDS scales therefore do not simply reflect integrated sensitivity, as has been recently suggested (Zhou et al., 2024), because they differentiate between transducers that produce identical integrated sensitivity.

### Generalizability of parameters

In the present simulation, we tested a wide range of transducer shapes to include compressive, linear, and expansive functions (power-law exponents α from 0.33 to 3.0). Most of the empirically estimated transducer functions in the visual domain are compressive or linear. Estimates from direct scaling have exponents ranging from 0.33 to 1.7 (Gescheider, 1997; Stevens, 1975). Functions for contrast (Shooner & Mullen, 2022) and luminance increments (Whittle, 1992) have been reported to be compressive (α ∼ 0.6 and 0.33, respectively), whereas for apparent line length, they are close to linear (Gescheider, 1997). Expansive functions are less common but have been reported for the perception of slant-from-texture (Aguilar et al., 2017) and for perceived image quality as a function of JPEG compression (Charrier, Maloney, Cherifi, & Knoblauch, 2007). The estimation bias we observed for expansive functions therefore does not seem to be particularly relevant in empirical practice, at least in the domain of low-level vision. However, if such a bias occurs, it can be recognized as a dip in the estimated scale (i.e., negative values at low stimulus levels).

Our hypothesis is that this is due to the mismatch between the simulated multiplicative noise model—where the noise magnitude varies across the scale—and the constant noise model assumed by MLDS in estimation. MLDS estimates both transducer shape and noise magnitude simultaneously but assumes that the noise magnitude is constant across the scale. For multiplicative noise, this assumption is violated, and an incorrect noise magnitude is estimated. For the expansive transducers, this could result in an estimated noise magnitude that is globally too high for the low end of the scale. This, in turn, would lead the estimation to produce more pronounced curvature (less uncertainty; the “dip”) in the scale at this low end to compensate for the uncertainty. For compressive transducers, a similar problem does not seem to appear at the flat, high end of the scale, presumably because differences are, on average, larger relative to the noise. For additive noise, the noise magnitude is constant across the scale, matching the assumption, and can thus be correctly estimated. Therefore, the same “dip” pattern does not occur with additive noise.

We consider these “dips” an artifact of the estimation. The negative scale values have no realistic interpretation, as negative perceptual values are not meaningful. This dip should not be confused with the “dipper function” in contrast discrimination, where sensitivity is suddenly higher (thresholds lower) at some low pedestal contrasts (e.g., Legge & Foley, 1980). The dipper reflects an increase in sensitivity, whereas our dip occurs for stimuli that are clearly visible but not distinguishable. Experimentally, a situation like this should be avoided by choosing stimulus levels that are pairwise discriminable, which can be done in pilot testing.

The range of noise magnitudes we used in the simulation was chosen to reflect values observed in actual experiments (additive noise: σ from 0.05 to 0.2, multiplicative noise: *g* from 0.0165 to 0.6). For example, in contrast discrimination, Kontsevich et al. (2002) reported values for multiplicative noise that ranged from 0.16 to 0.41 for a transducer that ranged from 0 to 1. A reanalysis of the same data by Georgeson and Meese (2006) showed that the data could also be fit by models with additive noise on the order of 0.03 to 0.1. For contrast appearance, Shooner and Mullen (2022) reported additive noise values around 0.1 to 0.2 in an MLDS experiment.

For the simulated Weber fractions, we also chose values that have been observed in different perceptual domains. For brightness, Weber fractions are reported to be around 8% to 10%, for line length around 4%, and for visual position 2% (a more complete list can be found in Baird (1978) and Teghtsoonian (1971)).

### Practicality of design and assumptions

We chose simulation parameters so as to consider design constraints in realistic experimental scenarios. We used 10 equidistant stimulus levels, which resulted in 120 unique triadic combinations for comparison judgments. Each comparison was repeated 10 times, so that each simulated MLDS experiment had 1,200 trials. In our experience, this typically corresponds to two 1-hour sessions for naive observers.

One can include more stimulus levels to provide more estimates for the underlying function. However, adjacent stimulus levels need to be distinguishable; otherwise, comparisons become more difficult, introducing more noise. MLDS is designed as a method for suprathreshold comparisons; hence, stimuli need to be spaced such that observers can sort them (Knoblauch & Maloney, 2012). The stimulus range, and hence the resolution limit for the scales, should in practice be determined in pilot testing. We suggest running simulations with putative ground-truth functions prior to an experiment to evaluate whether it is worth including more stimuli or more repeats. Such ad hoc ground-truth functions are of course an approximation, because they are the object of study and thus a priori unknown. Yet, simulation can help to decide on details of the experimental design. Our simulation code can be used for this purpose, available online (https://github.com/computational-psychology/aguilar_maertens_vincent_2026).

Our simulated observer follows the MLDS model, whereas real observers might not. MLDS attributes all variability in the judgments to a single noise source at the decision stage (Equation 8). In this model, the observer is most uncertain when the difference between intervals is small. Uncertainty should not depend on a particular stimulus in a triad, because all stimuli are individually visible and pairwise distinguishable. Real observers, however, might identify individual stimuli and choose, for example, always the pair containing the brightest or the darkest one, irrespective of the perceived difference. Such a response strategy would violate the model assumptions, and the goodness-of-fit routines could help to detect these cases.

### Relating discrimination and scaling

Recently, Zhou et al. (2024) proposed a framework to link stimulus appearance and discriminability that rests on two assumptions. First, sensitivity, expressed by Fisher sensitivity, can be related to a transducer and a certain noise type; second, absolute judgments (i.e., average ratings of stimulus magnitude) are a reliable and accurate estimate of the transducer. We agree with the first and disagree with the second assumption (see Direct methods). To illustrate the differences in approach, our respective strategies are depicted in Figure 10. Both frameworks try to link experimental data from discrimination and appearance judgments to a hypothetical underlying transducer and associated noise.

**Figure 10.**
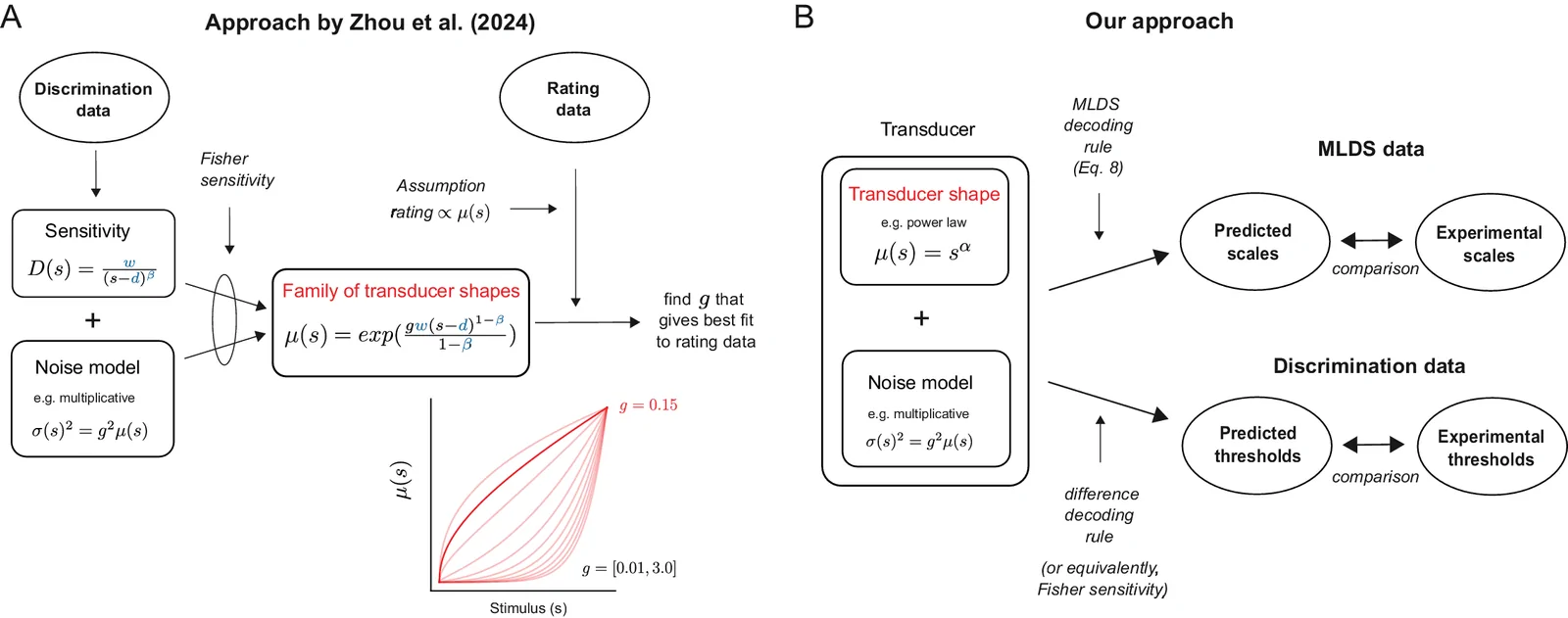
Relating discrimination and appearance. (**A**) Approach by Zhou et al. (2024). Different families of potential transducer shapes (red; mean percept μ(*s*)) correspond to different possible noise models (additive, multiplicative, Poisson, and modulated Poisson noise). These families are constrained by parameters (*w*, *d*, and β, in blue) estimated by fitting a generalized Weber’s law sensitivity function to empirical discrimination data. Additionally, the transducer shapes are determined by a free noise magnitude parameter (e.g., *g*; see inset plot). This free noise magnitude parameter and the other two remaining parameters (scale and shift of a linear transformation) are fitted to minimize squared error between the transducer and the empirical rating data. (**B**) In our approach, we choose all components of the generative model, that is, both the noise model (e.g., additive or multiplicative) and magnitude (e.g., σ or *g*), and the underlying transducer shape (e.g., logarithmic, or power law with exponent α). For each combination of noise and transducer, we simulate experimental data (scales, thresholds). This includes formulating an explicit rule on how to use the values on the transducer to simulate an observer’s decision (e.g., Equation 8 for MLDS). We have shown for the cases used here that from these simulated data, MLDS can estimate scales that reflect the transducer’s shape, mostly independent of additive or multiplicative noise model. Such simulated data can be compared to empirical data as a way of testing hypotheses about the underlying transducer and noise.

Zhou et al. (2024) reanalysed data from five different studies in three perceptual domains (visual, auditory, and gustatory) for which rating and sensitivity data were available. For each domain, they start from the empirical discrimination data and express it as Fisher sensitivity following a fitted generalized Weber’s law (Figure 10A). Then, for a potential noise model (additive, multiplicative, Poisson, and modulated Poisson noise), there is a corresponding family of transducer functions (μ(*s*)) that would produce the observed sensitivity. This family of transducers is constrained by the fitted sensitivity parameters, leaving a free noise parameter (inset plot). They then estimate this noise parameter by fitting the transducer to the empirical rating scale.^2^

Critically, Zhou et al. (2024) assume the mean rating data reflect the transducer. The rating scales, and hence transducers, were linear or compressive across the five studies used in their analyses (figure 4 in Zhou et al., 2024). In all five studies, the model with modulated Poisson noise did the best job. The transducers’ shape depended on the amount of noise (as seen in Figure 10A), and so the noise parameters varied widely across studies (*g* = 0.001 to 58, their figure 4).

We do not see a straightforward way to scale their or our transducers in order to quantitatively compare our respective parameters. But the main point of difference in argument concerns the question, What is the best method to estimate the transducer? We give preference to MLDS based on our above reasoning and based on our simulations. MLDS comes with an explicit observer model, which specifies how the “readings” on the transducer functions are used to derive a perceptual decision. It also explicitly incorporates noise. This is not the case for direct scaling methods. For discrimination and detection, the issue of noise, its sources, and their effect on the perceptual representation and decision, is an active area of study (e.g., Malo et al., 2025). One could use simulation to test different observer models in combination with direct scaling procedures, but to do so requires establishing a decision rule to generate the rating data. To us, it is not obvious which direct scaling models would be interesting to test. We are aware of only one recent study that has made an experimental comparison between direct and difference scaling. Devinck and Knoblauch (2023) compared direct estimation with maximum likelihood conjoint measurement (a method related to MLDS) for color appearance and found that both methods gave similar results. The assumptions behind direct scaling may thus hold in some cases; our point is that they cannot be guaranteed or tested experimentally. Future research might combine direct and difference scaling methods, together with simulations and experimental data.

### The transducer as construct and the role of simulations

At the core of the current work is the assumption of a transducer function, that is, a hypothetical mapping between physical stimulus values and their perceptual correspondences. It has been a matter of debate to what extent near-threshold and suprathreshold perceptual tasks rely on the same perceptual representation and hence can be modeled with the same function. Transducer functions are theoretical idealizations insofar as they concern the mapping of local stimulus variations to perception. They are agnostic to the effect of context, because in experiments that probe sensitivity, stimuli are often presented in isolation. However, context can substantially alter the appearance of a local stimulus region, for example, its brightness in simultaneous contrast (Hering, 1920) or the Adelson (1995) checkerboard. There have been attempts to consider different transducer functions empirically (e.g., Allred, Radonjic, Gilchrist, & Brainard, 2012; Hillis & Brainard, 2007a; Zeiner & Maertens, 2014), but the number of theoretically possible context-modulated mapping functions is infinite. From that point of view, the concept of a single transducer function seems of little use, because in real life, stimuli are always embedded in a context.

The transducer function is also a mathematical abstraction that tries to address the philosophical question of the perceptual representation by defining it relative to the physical stimulus. The transducer can thus be regarded as a handle to the perceptual representation that can be tackled experimentally. For that conceptualization, it is relevant to know which of the different experimental paradigms (i.e., discrimination, direct and difference scaling methods) is most suitable to probe the transducer.

Several recent studies have related sensitivity measured in discrimination tasks and appearance measured with MLDS. Kingdom (2016) was first to propose that these two measurements could be combined to estimate how internal noise varies with stimulus intensity, provided that both rely on a common transducer. Kingdom illustrated this principle with Whittle’s partition-scaling and discrimination data (Whittle, 1986; Whittle, 1992).

In the following years, several authors have used MLDS to estimate scales and related them to discrimination data, for example, for slant-from-texture stimuli (Aguilar et al., 2017), the watercolor effect (Devinck & Knoblauch, 2012), color contrast (Knoblauch et al., 2020), gloss (Cheeseman et al., 2021), achromatic contrast (Shooner & Mullen, 2022), and brightness (Shi & Eskew, 2024). Some of these studies have found agreement between the two measures. Adding those findings and our simulation results, we could be tempted to conclude that MLDS is the method of choice to estimate the true transducer function and hence perceptual representation.

We do not think that the conclusion can be reached in the mathematical or empirical domain alone, but rather argue in favor of combining simulation and experiment. If we observe a disagreement between sensitivity and appearance measures, it seems easy to conclude that they rely on different representations. However, they could still rely on the same representation, but one task requires additional or more complicated decision processes, which might be associated with additional variability. Shooner and Mullen (2022) found an agreement between empirical and predicted JNDs only after adjusting the noise level for the two-alternative forced-choice task, which might reflect an extra amount of noise in that task. If we observe an agreement between sensitivity and appearance measures, then it seems straightforward to conclude that they rely on the same representation. But, again, the agreement might result from a combination of different representations and decision processes that coincidentally result in the empirical agreement. We advocate simulation because it requires making all assumptions explicit (Figure 10B). Here, we tested whether MLDS is able to recover the transducer from psychophysical data, but the approach can be extended to include other psychophysical methods and transducer and noise models. Simulations are limited by the imagination of the experimenter as they test the generative models we experimenters come up with. But we can inject all that is known and generate quantitative predictions to compare with our data. It would be interesting to find a perceptual domain in which rating scales and MLDS yield different estimates for the transducer and compare them with measurements of discrimination thresholds. This would provide the ultimate empirical test for the theoretical relationships discussed here.

## Supplementary Material

Supplement 1

## Footnotes

1 Fechner originally suggested to use method (3) in a different way. He asked observers to repeat the adjustment multiple times and calculated the mean error of the settings to get an estimate of the difference thresholds.

2 Two additional parameters, corresponding to linear scale and shift factors, are required to bring the transducer and rating data into a common range.

## Appendix A. Appendix A: Deriving power-law transducer parameters that produce identical sensitivity

We aimed to simulate transducers that produced Weber’s law, that is, where JNDs (Δ*s*) are proportional to the stimulus value (*s*) by a constant proportionality factor. This proportionality factor is known as the Weber fraction (*k*), and Weber’s law can be expressed as

(12) $$\begin{array}{c} \Delta s=k\cdot s.\; \end{array}$$

Sensitivity is defined as the inverse of the JND. For Weber’s law, sensitivity *D*(*s*) is thus

(13) $$\begin{array}{c} D\left(s\right)=\frac{1}{\Delta s}=\frac{1}{k\cdot s}.\; \end{array}$$

How a transducer predicts sensitivity has been formalized in different ways in the literature (e.g., Kingdom & Prins, 2016; Shi & Eskew, 2024). We consider the most recent formalization by Zhou et al. (2024) called Fisher sensitivity. This measure is based on optimal efficient discrimination in a Fisher information sense. Fisher sensitivity depends on both the mean of the transducer μ(*s*) and its variance σ(*s*) produced by a stimulus *s*:

(14) $$\begin{array}{c} D\left(s\right)=\frac{|\mu^{'}\left(s\right)|}{\sigma (s)},\; \end{array}$$

where μ′(*s*) is the first derivative of the transducer μ(*s*).

By taking this definition of sensitivity, we can express Equation 13 as

(15) $$\begin{array}{c} D\left(s\right)=\frac{1}{k\cdot s}=\frac{\mu^{'}\left(s\right)}{\sigma (s)}.\; \end{array}$$

Then, taking the power-law transducer definition (Equation 3) and replacing μ(*s*), we have that

(16) $$\begin{array}{c} \sigma \left(s\right)=ks\mu^{'}\left(s\right)=ks\left(\alpha s^{\alpha -1}\right)\; \end{array}$$

(17) $$\begin{array}{c} =ks\left(\alpha s^{\alpha }s^{-1}\right)=k\alpha s^{\alpha }\; \end{array}$$

(18) $$\begin{array}{c} =k\alpha \mu (s).\; \end{array}$$

Thus, in order to obtain Weber’s law under a power-law transducer, the noise function σ(*s*) must be multiplicative, and the multiplicative factor is *k*α (compare to Equation 6):

(19) $$\begin{array}{c} g=k\alpha .\; \end{array}$$

In other words, transducers with different shapes (determined by their exponents α) can yield identical Weber’s law sensitivity at different noise magnitudes *g*, as long as the fraction $\frac{g}{\alpha }$ is kept constant.

## Appendix B: Simulation parameters and Weber fractions

Table A1 shows the simulation parameters for power-law transducers with multiplicative noise. These parameters were chosen in a way that four different Weber fractions could be produced for all different transducer shapes (controlled by parameter α).

**Table A1.** Simulation parameters for power-law transducers with multiplicative noise. The values in each cell are the Weber fraction (*k*) produced by a combination of the exponent parameter α and the parameter controlling the amount of multiplicative noise (*g*). Cases of equal Weber fraction are highlighted with gray backgrounds.
