# Supplementary material for: Using simulation to disambiguate transducer and noise
Source: J Vis. 2026 Oct 13;26(10):12. doi: 10.1167/jov.26.10.12 (PMC13676231; doi:10.1167/jov.26.10.12)
Supplement: Supplementary file 1 [file jovi-26-10-12_s001.pdf]

Supplementary Material

Using simulation to disambiguate transducer and noise

Guillermo Aguilar*[1], Marianne Maertens†[1], and Joris Vincent‡[1]

[1]Computational Psychology, Technische Universität Berlin, Berlin, Germany,
https://www.psyco.tu-berlin.de/

1 Bias of MLDS estimation

We explored the distribution of the errors between the estimated perceptual scale and the ground-truth transducer, specifically to see whether MLDS is biased in its estimation. Errors were calculated as the signed difference between the estimated scale value and the ground-truth transducer (Eq. 10 of the main text).

Fig. S1 and S2 show the distribution of errors as a function of the stimulus intensity, for additive and multiplicative noise, respectively. For additive noise, errors are distributed uniformly across stimulus intensity, not showing bias. As expected, errors increase with the simulated noise magnitude. A higher bias was observed for multiplicative noise. In general for the compressive transducers (particularly the logarithmic) errors are on average positive, meaning the MLDS scales are overestimating the transducer. This bias is more pronounced for lower stimulus values, thus the scales are overestimating the transducer more in this range. For the expansive transducer, MLDS tends to underestimate the transducer, and this bias is also more pronounced for lower stimulus values. For additive noise (Fig. S1), errors increase with the simulated noise magnitude, as expected.

*Corresponding Author: guillermo.aguilar@mail.tu-berlin.de
†marianne.maertens@tu-berlin.de
‡joris.vincent@tu-berlin.de

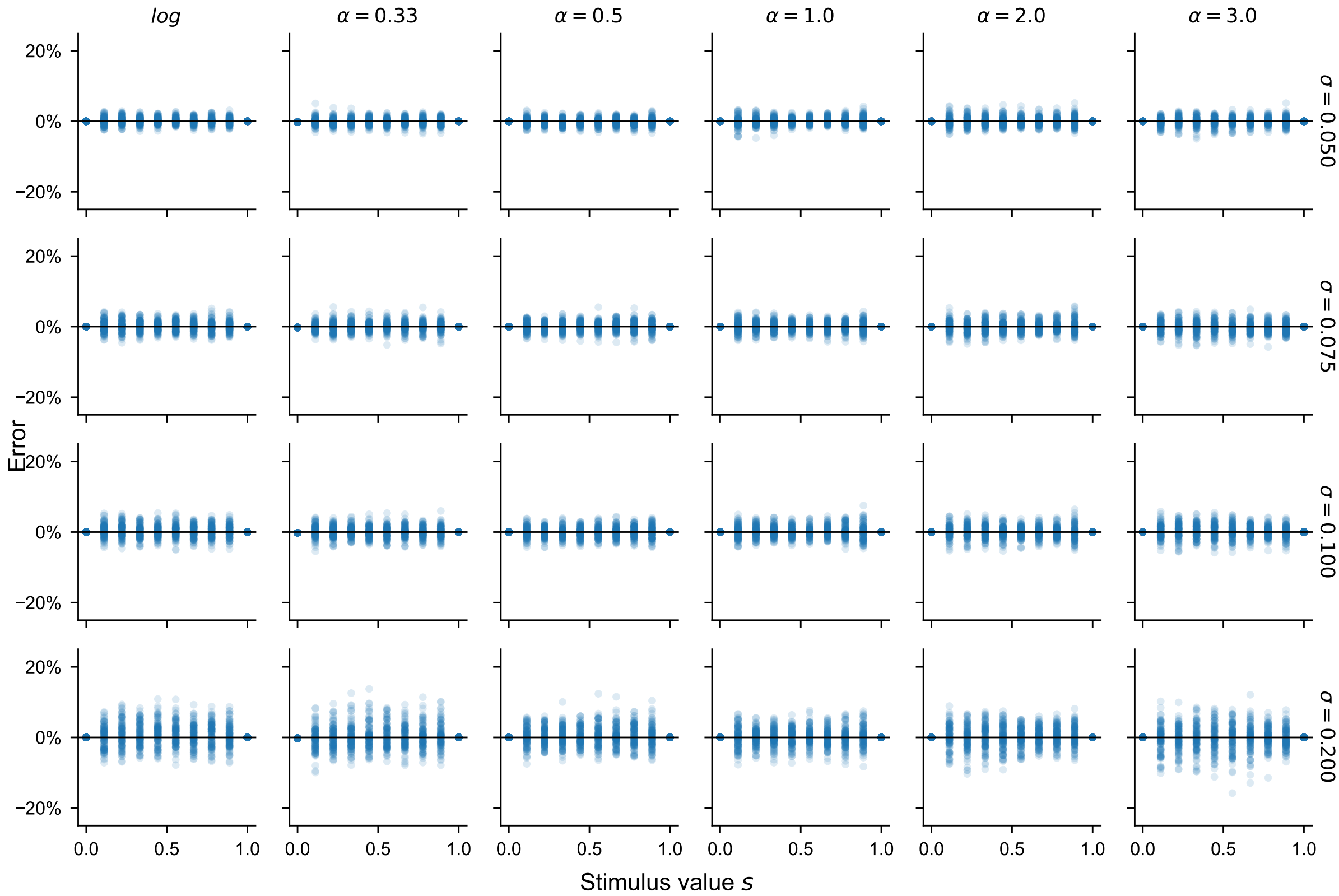

Figure S1: Distribution of errors in the estimation of power-law transducer functions with additive noise. Error is the signed difference between the estimated perceptual scale and the ground-truth transducer function.

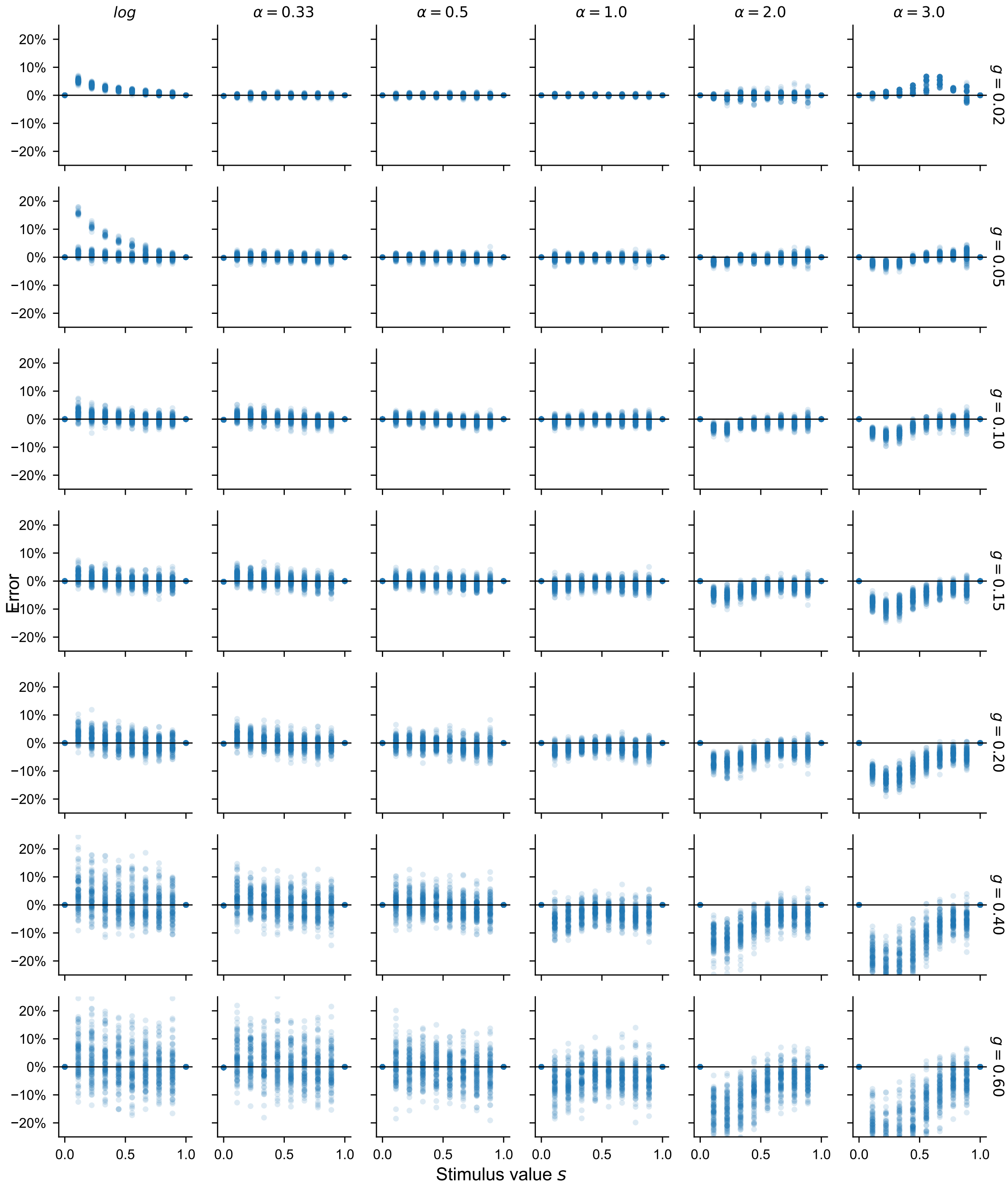

Figure S2: Distribution of errors in the estimation of power-law transducer functions with multiplicative noise. Error is the signed difference between the estimated perceptual scale and the ground-truth transducer function.

2 Poisson noise

In the main text we modeled the perceptual representation as a Gaussian random variable whose noise magnitude was either constant (additive noise) or proportional to the transducer's mean (multiplicative noise). Both noise distributions are symmetric. A physiologically motivated alternative is Poisson noise, in which the internal representation is a count whose variance equals its mean (Goris, Movshon, & Simoncelli, 2014; Zhou, Duong, & Simoncelli, 2024). Poisson noise differs from the additive and multiplicative cases in three respects that are relevant for MLDS: the noise magnitude grows with the square root of the transducer's mean rather than in direct proportion to it, the distribution is asymmetric, and it is discrete. Since MLDS assumes a Gaussian decision variable of constant variance, all three are violations of its model assumptions. Here we simulate transducers with Poisson noise and explore whether MLDS can also estimate their shape for this case.

Methods

Poisson noise is a discrete distribution, often used to represent the number of events occurring in a fixed interval of time or space, such as 'spikes' in a neural population. This has a different distribution than Gaussian noise, mainly because it is not symmetric and has a lower bound at zero. Like multiplicative noise, Poisson noise is variable noise as its magnitude increases with stimulus value. Unlike multiplicative noise, it has a fixed relationship between mean and variance:

$$\sigma^2(s) = \mu(s) \tag{S1}$$

Then perceptual representations are modeled not as a Gaussian random variable (as in Eq. 2 of the main text) but as Poisson,

$$\psi(s) \sim \text{Poisson}(\mu(s)) \tag{S2}$$

We simulated Poisson noise for the same logarithmic and power-law transducers as for the other noise types. To correctly simulate them, however, we had to introduce two changes to our methods: i) scale the range of the transducer and ii) slightly modify the decision rule to account for the case where the decision variable is exactly zero, which happens often with this noise. In the following we clarify these two modifications.

The transducers were defined over the range [0, 1]. For a Poisson distribution this implies that its mean is never greater than 1. In this range, the Poisson distribution is coarsely discrete: at the top of the range it draws either 0 or 1 with probability 0.74, and 2 or more with probability 0.26. Furthermore, Poisson noise is not invariant to a rescaling of the internal axis, unlike Gaussian noise, so expressing the transducer on [0, 1] also fixes the noise magnitude, at the largest value this noise model allows. Thus, to have a graded noise magnitude we introduced a scaling parameter c, which increases the mean of the Poisson distribution,

$$\psi(s) \sim \frac{1}{c}\text{Poisson}(c \cdot \mu(s)) \tag{S3}$$

giving $\sigma(s) = \sqrt{\mu(s)/c}$. One interpretation of this scaling parameter is that it links the transduced perceived magnitude to a neural firing rate modeled by the Poisson process. The value $1/\sqrt{c}$ corresponds to the noise magnitude at the maximum transducer value ($\mu(s) = 1$), which is the same quantity denoted by σ for additive and by g for multiplicative noise. We therefore chose c so that this noise magnitude takes the values 0.1, 0.2 and 0.6 ($c = \{100, 25, 2.78\}$), to make simulations comparable with the ones

with additive and multiplicative noise. In addition we simulated $c = 1$, which gives a noise magnitude of 1, the largest this noise model can produce on these transducers. Note that the scaling parameter c also affects the shape of the noise distribution: a lower value of c produces a more asymmetric noise.

The second modification concerns the decision rule. As the representation is now discrete, the decision variable is exactly zero on a substantial fraction of trials. With the original decision rule in Eq. 9 of the main text such cases were always assigned to one response (choosing the lower pair (s_1, s_2)), although they never occurred. With Poisson noise, however, these cases occurred often and we needed to handle them explicitly. We resolved these cases by assigning the response at random, that is, whenever the decision variable was exactly zero ($\Delta = 0$ in Eq. 8 of the main text) we assigned either response with probability 0.5. This corresponds to an observer that guesses when the two pairs of stimuli have exactly the same perceptual difference.

Note that these transducers do not produce Weber's law sensitivity with Poisson noise. Therefore, none of these simulations are part of the subset of simulations that produce identical sensitivity.

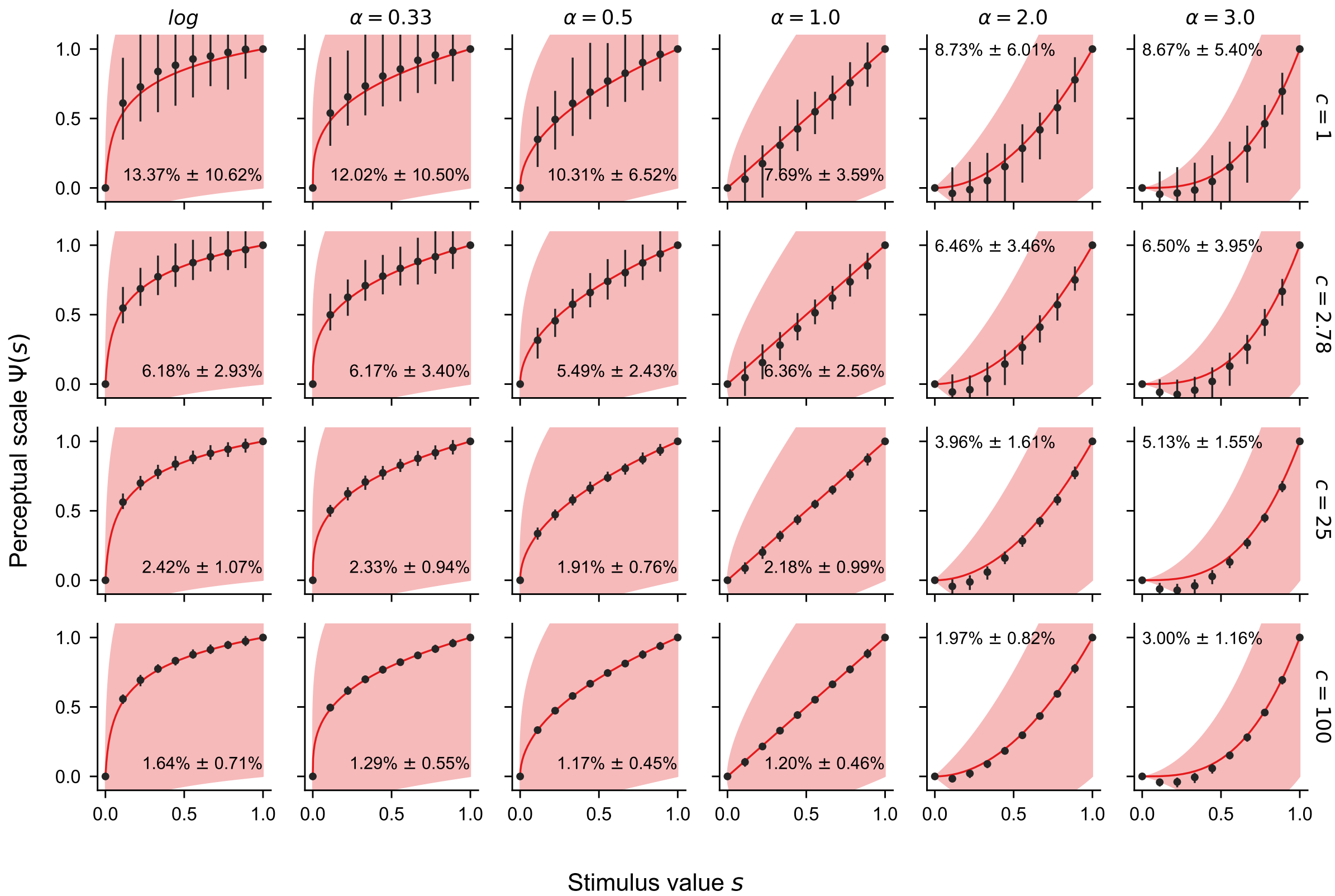

Figure S3: **Simulation results for transducers with Poisson noise.** Analogous to Figs. 5 and 6 of the main text.

Results

MLDS recovers the shape of the transducer at all four noise magnitudes (Fig. S3). RMSE ranged from 1.2–3.0 % at $c = 100$ up to 7.7–13.4 % at $c = 1$, where the noise standard deviation at the top of the range equals the whole range of the transducer. As for the other noise types, the error grows with noise magnitude (Fig. S4).

For the compressive and logarithmic transducers the scales overestimate the transducer at low stim-

ulus intensities, and for the expansive transducers they underestimate it, producing the same 'dip' as for the multiplicative noise simulations (Fig. 6 of the main text).

Fitting a power law to each estimated scale recovers the ground-truth exponent with an overestimation that grows with the exponent (Fig. S5). The median estimate is within 6.1 % of ground truth at $c = 100$, and within 19.2 % at the larger noise magnitudes.

We conclude that MLDS is also able to estimate the transducer's shape with Poisson noise, in a similar way as with multiplicative noise, although with a smaller bias for the expansive transducers.

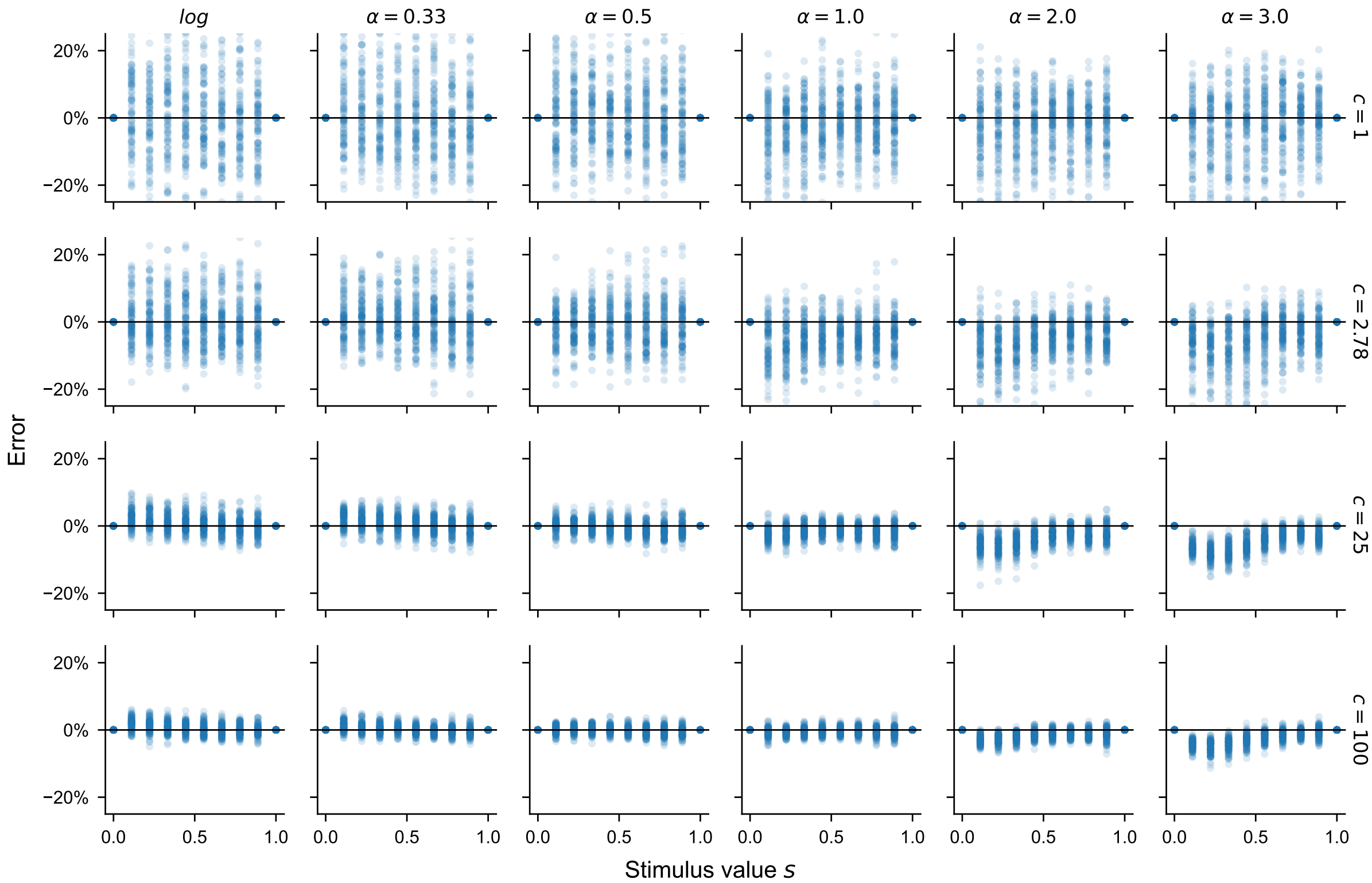

Figure S4: Distribution of errors in the estimation of power-law transducer functions with Poisson noise. Error is the signed difference between the estimated perceptual scale and the ground-truth transducer function.

References

Goris, R. L. T., Movshon, J. A., & Simoncelli, E. P. (2014, June). Partitioning neuronal variability. *Nature Neuroscience*, *17*(6), 858–865. doi: 10.1038/nn.3711

Zhou, J., Duong, L. R., & Simoncelli, E. P. (2024, June). A unified framework for perceived magnitude and discriminability of sensory stimuli. *Proceedings of the National Academy of Sciences*, *121*(25), e2312293121. doi: 10.1073/pnas.2312293121

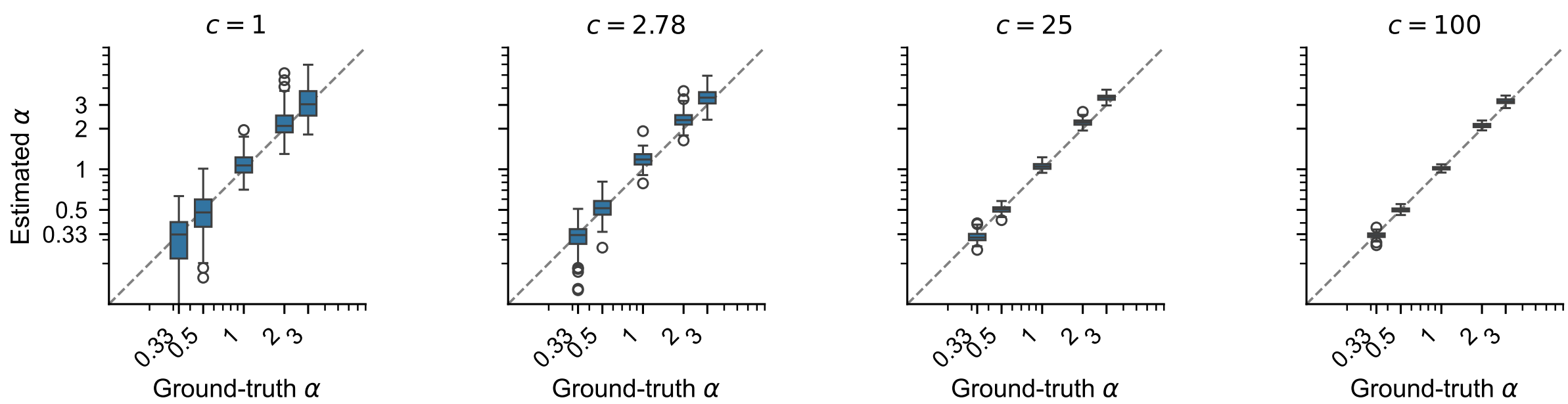

Figure S5: **Recovery of the transducer exponent α from MLDS scales**. The estimated exponents (y-axis) were obtained by fitting each simulated scale to a power-law function. The boxplots show the distribution of the estimates across 100 simulations, run for different transducer shapes (x-axis), and different scaling parameters for the Poisson noise. The diagonal dashed line indicates the identity line.
